# Alexithymia in Patients with Somatization Difficulties and Tinnitus-Related Distress: A Systematic Review

**DOI:** 10.3390/jcm12216828

**Published:** 2023-10-29

**Authors:** Dominic Freiherr von Schoenhueb, Benjamin Boecking, Birgit Mazurek

**Affiliations:** Tinnitus Center, Charité—Universitatsmedizin Berlin, 10117 Berlin, Germany; dominic.schoenhueb@charite.de (D.F.v.S.); benjamin.boecking@charite.de (B.B.)

**Keywords:** alexithymia, chronic tinnitus, tinnitus-related distress, somatoform conditions, somatization, psychotherapy

## Abstract

Chronic tinnitus, the perception of sound without an external source, can significantly affect individuals’ well-being. As an often medically unexplained symptom, chronic tinnitus can present as a “somatoform” or “functional” difficulty. Some evidence has pointed to alexithymia as a transdiagnostically relevant risk factor for both symptom clusters. Using a two-part rapid review—searching within EBSCO, Embase by Ovid, PubMed, Web of Science—we summarize psychological studies regarding alexithymia, i.e., difficulties in recognizing and expressing emotions and (1) somatoform conditions and (2) chronic tinnitus. For the former (inclusion criteria: (1) adult human beings with different kinds of somatization, (2) longitudinal study designs, (3) publication between 2001 and 2021, (4) full-text in English or German) we identified eight studies that revealed significant links between alexithymia and somatoform conditions. Psychotherapy improved alexithymia in most studies. Additionally, alexithymia was associated with broader treatment outcomes such as improvements in pain intensity, gastrointestinal symptoms, and patient-therapist alliance. The ‘Risk Of Bias In Non-randomized Studies—of Interventions’ tool (ROBINS-I) and ‘Revised Cochrane risk-of-bias tool for randomized trials’ (RoB 2) were used for risk of bias assessment. Summarizing all available studies on alexithymia and chronic tinnitus, we identified three studies. Inclusion criteria were: (1) adult human beings with chronic tinnitus, (2) publication between 2001 and 2021, (3) full-text in English or German. Risk of bias was assessed by the ‘JBI Critical Appraisal Checklist for Analytical Cross Sectional Studies’. The available studies suggested a high rate of alexithymia (65.7%) in patients with chronic tinnitus. Tinnitus-related distress was significantly associated with alexithymia in two studies, one of which, however, found no differences in alexithymia between patients with bothersome versus non-bothersome tinnitus. Conversely, one study reported high levels of alexithymia in patients with low levels of tinnitus-related distress. Overall, alexithymia may be a transdiagnostic psychological indicator of somatization phenomena, which might include some chronic tinnitus presentations. Psychotherapy likely improves alexithymia as well as somatoform symptom presentations.

## 1. Introduction

Somatization denotes the occurrence of bodily expressed symptoms which cannot be explained completely or adequately by organic causes [1]. Lloyd [2] defined somatization as “presentation of psychological distress by way of somatic complaints”; thereby, he operationalized somatization by different characteristics: (a) presenting somatic symptoms belonging to pervasive emotional distress, (b) attribution of both physical symptoms and experienced emotional distress to physical causes, (c) the absence of identifiable physical pathology, and (d) a process that occurs within the overall context of pervasive emotional distress. Somatization can thus be seen as an expression of psychological distress in a somatic way [3].

Defining somatization as a process wherein psychological distress is reflected or expressed in a somatic manner, it is a transdiagnostic process across different diagnostic categories used in traditional psychiatric nosologies. In contrast to assumed distinct psychiatric disorder categories, transdiagnostic approaches cross such boundaries, focusing on underlying factors of different symptom expressions instead [4]. To this regard, Witthoft [5] points out the relevance of transdiagnostic approaches in understanding somatization and somatoform symptoms beyond diagnostic categories.

In attempting to understand somatization, studies repeatedly highlight the importance of personality constructs. Amongst such factors of interest, reviews have concluded associations between somatization and neuroticism (i.e., a “tendency to experience frequent, intense negative emotions associated with a sense of uncontrollability (the perception of inadequate coping) in response to stress” [6]) as well as [7], self-defeating [7], harm-avoiding [7] and somatosensory amplification styles (Somatosensory amplification describes “the tendency to experience somatic sensation as intense, noxious, and disturbing” [8]) [9].

In recent years, research efforts began to examine alexithymia as a possible contributor to somatization. Alexithymia comprises difficulties in identifying, describing, and distinguishing emotions as well as an externally oriented style of thinking [10,11]. 

Whilst alexithymia has garnered increased attention as a personality trait and potential risk factor for somatization [12,13,14,15] within broader vulnerability–stress models, no study has yet reviewed the available evidence to this regard. Considering some forms of chronic tinnitus as the potential result of somatization processes, investigating the role of alexithymia in maintaining chronic tinnitus seems to be important. The current review thus examines (a) associations between alexithymia and different kinds of somatoform presentations as well as (b) the relation between alexithymia and chronic tinnitus.

### 1.1. Alexithymia

Contemporary definitions view alexithymia as a multidimensional psychological construct, which includes difficulties in recognizing, identifying, and describing feelings as well as in differentiating between emotions and bodily symptoms [16,17]. In the psychological literature, alexithymia is defined as both (a) a personality trait, and as (b) a resultant state of depressivity, anxiousness, chronic psychopathology, and somatization [13]. As a trait, alexithymia exists on a normally distributed continuum within the general population [18,19]. Expressions of alexithymia in the general population appear to differ regarding (a) gender—men seem to show higher expression [20,21]; (b) increasing age, meaning the older a person is the harder it is to identify and/or describe emotions [20,22]; (c) educational level—the lower the level, the higher the alexithymia [20,22]; (d) perceived health—the lower the health, the higher the alexithymia [20]; and (e) “depression” [20].

More broadly, alexithymia appears closely associated with a variety of psychological constructs including anxiety, depression, social functioning, and somatization [23]. Evidence suggests that alexithymia is a risk-factor that may transdiagnostically contribute to different forms of psychopathology [24]. Gaggero et al. [25] conducted a review of the history of alexithymia and described alexithymia as a non-specific vulnerability factor for disorders related to emotional dysregulation as well as personality disorders. Other authors discussed alexithymia as a possible risk factor for increased vulnerability for organic diseases, maladaptive lifestyle behaviors, and a biased perception and reporting of somatic sensations and symptoms [26,27]. The limited emotional awareness and cognitive affect processing may lead to a “prolonged and amplified physiological arousal and neurovegetative reactivity to stress, thus potentially disturbing the autonomic, pituitary–adrenal and immune systems” [13]. Martínez-Sánchez et al. [13] view this dysregulation and/or increased activation of the autonomic nervous system as key factor in explaining associations between alexithymia and a proneness to functional somatic disorders.

### 1.2. Alexithymia and Somatization

Different authors reported associations between alexithymia and somatization [23,28,29,30,31,32,33,34,35,36,37]. Overall, the prevalence rates of alexithymia in patients with pervasive emotional difficulties is high, and alexithymia may pose a transdiagnostic vulnerability factor for such difficulties [38]. The highest prevalence rates of alexithymia were found in patients with psychotic, depressive, somatoform, and anxiety-related difficulties [39]. Additionally identified associations linked alexithymia to the number of presented physical symptoms in clinical contexts [31,33,37,40,41], negative affect [33] and somatic symptom severity [42].

At the extreme end of the spectrum, somatoform conditions denote psychiatric classifications, which lead to the expression and experience of pervasive bodily symptoms due to high levels of psychological distress. Persistently expressed physiological symptoms suggest an underlying medical condition to patients and practitioners—although the symptoms cannot be adequately explained by a medical condition as such [43]. 

Within diagnostic classification systems, the phenomenon is descriptively coded as ‘Somatic Symptom Disorder’ (SSD) [43]. SSD is defined by criteria shown in Table 1. 

The concept of somatoform disorders is discussed controversially and a distinction between somatic symptom disorders and functional syndromes is made [44]. In light of such controversies, a transdiagnostic perspective may all the more helpful in placing emotional experiences at the heart of medically unexplained symptom presentations [45].

Alexithymia is commonly overserved in patients with somatoform conditions [17,46], anxiety [47], or depression [17,47]. In addition, higher levels of alexithymia are associated with higher rates of comorbidity diagnoses [48], suggesting a transdiagnostic role across various cognitive-affective-behavioural difficulties [49,50]. More specifically, it has been argued that psychological conditions may not emerge from an ‘absence of affect’ but from its ‘undifferentiated structure’ [49]. To this regard, Muehlenkamp et al. [51] suggest that patients with few emotion-regulation skills may have no other way to regulate their emotional states, than to develop medically unexplained symptom presentations. 

### 1.3. Measurement of Alexithymia

Alexithymia is commonly assessed using the ‘Toronto Alexithymia Scale’ (TAS) [52,53], which has been translated into different languages and validated in different cultures [54,55,56,57,58,59,60,61]. The TAS allows calculating a total score as well as three subscale scores named (1) Difficulties in identifying feelings (DIF), (2) Difficulties in describing feelings (DDF), and (3) Externally-oriented thinking (EOT). Each item is rated on a 5-point Likert-scale from 1 = strongly disagree to 5 = strongly agree.

Kajanoja et al. [47] suggested two subtypes of alexithymia—type A and type B—following cluster analysis of results from a study with *N* = 2876 participants. Type A was characterized by high difficulties in describing feelings (DDF) and externally oriented thinking (EOT), whereas type B was characterized by difficulties in identifying feelings (DIF) [47]. In their work, the authors suggested that higher prevalence rates of depressive and anxiety-related phenomena were associated with type B alexithymia [47].

### 1.4. Alexithymia and Psychological Treatment

Alexithymia influences both therapeutic process as well as treatment outcomes [62,63]. For example, higher levels of alexithymia at baseline were found to be associated with poorer outcomes of psychotherapy [64,65,66]. Importantly, however, alexithymia was also found to be modifiable by therapeutic interventions [67,68,69,70,71,72], thereby constituting a viable psychological treatment target. Taylor [73] points out that psychotherapy—which is adapted to one’s individual needs and difficulties with emotional awareness—may effectively reduce alexithymia and increase psychological well-being. These thoughts were supported by Grabe et al. [74], who reported that psychodynamic group therapy, which focused on facilitating awareness of emotion, significantly improved alexithymia as measured by TAS-20. CBT was also found to significantly decrease alexithymia scores [75,76].

### 1.5. Chronic Tinnitus

Chronic tinnitus (lasting for more than 3 months [77]) is a common symptom which is defined as the persistent perception of a sound without the presence of an external acoustic source [78]. For chronic presentations, psychological influences play a crucial role in symptom onset and maintenance [79]. De Ridder et al. [80] point out that even if acute tinnitus initially onsets due to a somatic cause, chronic tinnitus is currently seen as an independent, distress-related phenomenon. For chronic tinnitus, differences in case-control studies show abnormalities in attention, memory, and limbic systems, which may equally be attributable to psychological mechanisms [81,82]. Consequently, chronic tinnitus may be seen as a somatoform complaint [83].

Whilst chronic tinnitus is not yet defined as a “somatoform condition” by the World Health Organization [84], it can be viewed as a medically unexplained, distress-related symptom—and thus a potential somatization phenomenon. Hiller et al. [85] suggest three possible pathways that may reflect the relation between chronic tinnitus and somatoform conditions: First, chronic tinnitus may be an independent somatoform condition; second, chronic tinnitus and somatoform conditions may be frequently “comorbid”; and third, both chronic tinnitus and somatoform symptom presentations may share underlying psychological or autonomic processes. Here, potential construct overlaps ought to focus on the shared variance of risk factors and psychological expressions of emotional distress. For example, research has begun to identify close links between the phenomenologies of chronic tinnitus and chronic pain experiences, with both symptom clusters being closely associated via psychological factors, likely explaining shared underlying variance [86,87].

In a recent systematic review, Kleinstäuber and Weise [88] postulated that a combination of emotional distress and somatization tendencies predicted the development of “bothersome” chronic tinnitus. Tinnitus becomes subjectively more bothersome at times of emotional distress [89,90] and is consequently closely linked to psychological, not audiological factors “per se” [79,91]. Similarly, Probst et al. [92] reported that the relation between perceived tinnitus loudness and tinnitus-related distress was partially mediated by emotional factors such as arousal and valence [92]. 

Thus, it may be possible that an acutely perceived sound becomes chronic via existing as well as reactive psychological distress against the background of both alexithymia and somatization tendencies as personality predispositions [93].

The phenomenology of chronic bothersome tinnitus often overlaps with other psychological phenomena known to correlate with alexithymia such as depressivity and anxiety [94,95,96,97,98,99], social anxiety [95], obsessionality [83] and other somatoform symptom presentations [96,98,100,101]. Overall, existing psychological distress strongly influences the interpretation, evaluation and persistence of the tinnitus symptom, thereby facilitating chronification [94].

Given the substantial overlap of chronic tinnitus, its bothersomeness, and other frequent distress-related psychological phenomena, Kratzsch and Goebel [102] state that comorbid psychological factors need to be taken into account for any planning of treatment. Even more poignantly, Zirke et al. [103] as well as Boecking et al. [104] ask to primarily focus on psychological phenomena in the treatment of chronic tinnitus. Indeed, psychological treatment approaches constitute the gold standard treatment for patients with chronic tinnitus [105]. This observation is both crucial for the development and advocacy of adequate treatment pathways for patients with chronic tinnitus and strongly under-implemented, given that psychological interventions are just recommended to 0.2% of affected patients [106].

### 1.6. Chronic Tinnitus and Alexithymia

Given the co-occurrence of chronic tinnitus and other somatization symptoms against a backdrop of personality-related vulnerability factors as well as pre-existing depressivity or anxiety, respectively, surprisingly few studies investigated associations between alexithymia and chronic tinnitus—although one study found the prevalence of pronounced alexithymia to be as high as 65.7% in a sample of patients with chronic tinnitus [107]. Both Bakhla et al. [107] and Wielopolski et al. [108] observed positive correlations between subjectively reported tinnitus-related distress, measured by Tinnitus Handicap Inventory (THI), and alexithymia. Bakhla et al. [107] suggest that higher tinnitus-related distress may be associated with difficulties in describing or identifying emotions—likely in those individuals who are vulnerable to react to the tinnitus sound in a distressed manner.

Salonen et al. [109] reported a correlation between alexithymia and the presence of tinnitus by using an individual tinnitus questionnaire but did not find a direct association between alexithymia and the annoyance of tinnitus.

Interoception (i.e., the “process by which the nervous system senses, interprets, and integrates signals originating from within the body, providing a moment-by-moment mapping of the body’s internal landscape across conscious and unconscious levels” [110]) is related to somatoform conditions. The poorer one’s interoceptive awareness, the more somatoform symptoms emerge [111]. Tinnitus seems also to be a result of interoceptional processes [112]. 

Flasinski et al. [113] found no significant distinction between healthy controls and patients suffering from somatoform conditions in the “awareness of noticing of interoceptive signal processing” but within distraction and self-regulation. Herbert et al. [114] also found that interoception is linked to other psychological aspects, such as alexithymia. Interoceptive awareness was inversely related to alexithymia and negatively associated with the EOT subscale in male subjects [114]. A meta-analysis revealed a negative correlation between alexithymia and interoceptive perception, although the strength of this correlation differed across underlying psychological conditions [115]. These results suggest interoception to be a transdiagnostically relevant process that underlies emotional processing across different clinical phenomena [115]—including alexithymia and chronic tinnitus. 

### 1.7. Objectives and Hypotheses

The primary aim of this review was to investigate whether, and if so, to what extent alexithymia affects therapeutic outcomes in patients with somatoform symptom presentations. The secondary aim was to summarize findings on alexithymia in patients with chronic tinnitus.

## 2. Methods

Reporting methods of the current systematic review is followed by the Preferred Reporting Items for Systematic reviews and Meta-analyses (PRISMA), initially developed and published by Moher et al. [116] and last updated by Page et al. [117].

We conducted two independent searches and review processes. The first review focused on alexithymia and somatoform conditions; the second, on alexithymia and chronic tinnitus. Differences and similarities within eligibility criteria of both review processes are displayed in Table 2.

### 2.1. Alexithymia and Somatoform Conditions

#### 2.1.1. Eligibility Criteria

To maximize inclusivity of identified studies, inclusion criteria comprised studies involving

- Adult human beings (≥18 years old) who showed different kinds of somatization. Studies investigating animal or child-or-adolescent samples were excluded.
- Assessment of both alexithymia and somatization with self-report questionnaires including a version of the Toronto Alexithymia Scale—TAS, TAS-R, TAS-20—published by Bagby, Parker and Taylor [52] and Bagby, Taylor and Parker [53] or by an equivalent self-report measure.
- A longitudinal or prospective study design. Cross-sectional or case-report studies were excluded.
- Publication between 2001 and 2021, as a previous review by De Gucht, Heiser and De Gucht [37] reported findings on the relation between alexithymia and somatization from between 1985 and 2000.
- Full-text availability in English or German. Conference papers, letters or guidelines were excluded.

#### 2.1.2. Search Strategy

The online research databases EBSCO, EMBASE by Ovid, PubMed and Web of Science were searched on 4 February 2022. EMBASE did not yield any results. To identify relevant records, the used search terms were specified by including key words and relevant synonyms of alexithymia, somatoform conditions, longitudinal study design as well as psychotherapeutic treatment. To identify relevant synonyms, the National Library of Medicine [118] was searched for Medical Subject Headings (MeSH) as well as already published reviews and studies within the field of research focusing on somatization and longitudinal therapeutic interventions. The identified MeSH were used in combination with relevant text words to define search terms for databases. The used search terms are displayed in Table 3. Additionally, reference lists of selected records were screened manually.

#### 2.1.3. Study Selection

**Figure 1:**
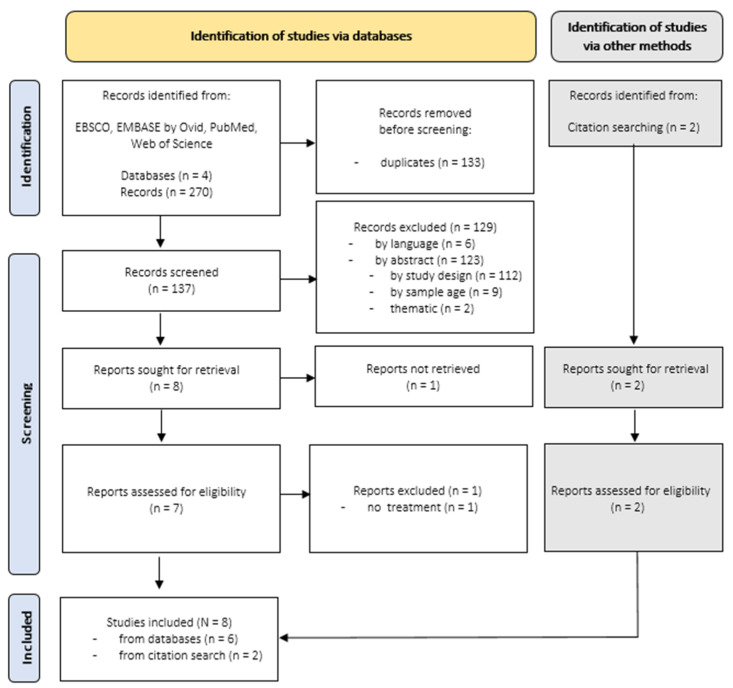
Search Results for Longitudinal Studies focusing on Alexithymia and Somatoform Conditions.

Overall, searches within selected databases identified a total number of 270 records published between 2001 and 2021, focusing on alexithymia and somatization. After removal of duplicates (*n* = 133), 137 records remained. The remaining records were first screened by titles, afterwards by abstracts and ultimately by full texts. One full text could not be retrieved [119]. The selection process is displayed in Figure 1. The whole selection process was undertaken by a single researcher, and uncertainties were resolved by discussion with an experienced research member. Two additional records were identified by reference lists [120,121]. Ultimately, eight longitudinal studies were included in this first part of the review.

#### 2.1.4. Data Extraction

Duplicates (*n* = 133) within identified records were removed. All records for which full texts were retrieved were entered in an Excel file containing the following information: (1) study-ID, (2) authors and year, (3) title, (4) measurement timepoints, (5) study outcome, (6) study outcome measurement, and (7) type of treatment. Finally, results were saved as PDF files. These results of data extraction can be found in Supplementary Table S1.

#### 2.1.5. Risk of Bias

We include both studies designed as randomized controlled trials (RCT) [121,122] and studies with non-randomized designs (NRCT) [71,120,123,124,125,126]. In order to assess risk of bias, we used the ‘Risk Of Bias In Non-randomized Studies—of Interventions’ tool ROBINS-I [127] as recommended by Ma et al. [128]. The ‘Revised Cochrane risk-of-bias tool’ for randomized trials RoB 2 [129] was used to assess risk of bias of the two included RCTs [128]. Risk of bias assessment was conducted by a single researcher. Uncertainties were resolved by discussion within the research team.

#### 2.1.6. Data Synthesis

Data were synthesized with regard to whether alexithymia affects therapeutic outcomes in patients with somatoform symptom presentations, and if so, to what extent. 

### 2.2. Alexithymia and Tinnitus

#### 2.2.1. Eligibility Criteria

To maximize inclusivity of identified studies, inclusion criteria were defined as

- Adult human beings (≥18 years old) suffering from chronic tinnitus. Studies investigating animal or child-or-adolescent samples were excluded.
- Assessment of both alexithymia and chronic tinnitus with self-report questionnaires including a version of the Toronto Alexithymia Scale—TAS, TAS-R, TAS-20—published by Bagby, Parker and Taylor [52] and Bagby, Taylor and Parker [53] or by an equivalent self-report measure.
- Alexithymia and chronic tinnitus needed to be assessed by self-report questionnaires. Therefore studies needed to asses alexithymia via one version of the Toronto Alexithymia Scale—TAS, TAS-R, TAS-20—published by Bagby, Parker and Taylor [52] and Bagby, Taylor and Parker [53] or by equivalent questionnaires.
- Case reports were excluded.
- Publications between 2001 and 2021.
- Full-text availability in English or German. Conference papers, letters or guidelines were excluded.

#### 2.2.2. Search Strategy

The online research databases EBSCO, EMBASE by Ovid, PubMed and Web of Science were searched on 4 February 2022. EMBASE did not yield any results. To identify relevant records, the used search terms were specified by including potentially relevant synonyms of tinnitus and alexithymia as keywords. To identify relevant synonyms, the National Library of Medicine [118] was searched for Medical Subject Headings (MeSH) as well as already published reviews and studies focusing on tinnitus. These identified MeSH were used in combination with relevant text words to define search terms for databases. Defined and used search terms are displayed in Table 4. In addition, reference lists of selected records were screened manually.

#### 2.2.3. Study Selection

**Figure 2:**
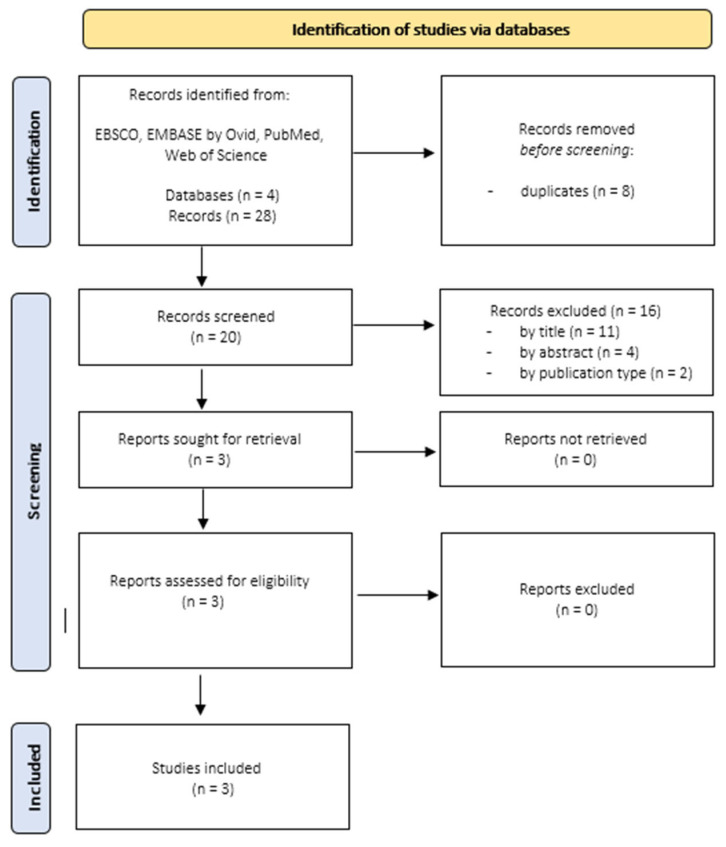
Search Results Studies focusing on Alexithymia and Tinnitus.

Focusing on alexithymia and chronic tinnitus, 28 records published between 2001 and 2021 were identified. When duplicates (*n* = 8) were removed, 20 records remained. Three records remained after screening for titles and abstracts. This selection process is displayed in Figure 2. 

#### 2.2.4. Data Extraction

Duplicates (*n* = 8) within identified records were removed. All records for which full texts were retrieved were entered in an Excel-file containing information like (1) study-ID, (2) authors and year, (3) title, (4) measurement timepoints, (5) study outcome, (6) study outcome measurement, and (7) type of treatment. Finally, results were saved as PDF files. Results of data extraction can be found in Supplementary Table S2.

#### 2.2.5. Risk of Bias

The assessments of risk of bias for the three included studies were carried out by the ‘JBI Critical Appraisal Checklist for Analytical Cross Sectional Studies’ [130]. We decided on this tool of assessment due to its recommendation for analytical cross-sectional studies by Ma et al. [128]. Risk of bias assessment was conducted by a single researcher. Uncertainties were resolved by discussion within the research team.

#### 2.2.6. Data Synthesis

The data were summarized with regard to findings on alexithymia in patients with chronic tinnitus. 

## 3. Results

### 3.1. Alexithymia and Somatization

#### 3.1.1. Study Characteristics

Eight studies met inclusion criteria for the first part of the current review (see Table 5). The majority of included studies were conducted in the European Region (5/8, 62.5%), followed by the Region of the Americas (2/8, 25.0%), and the Eastern Mediterranean Region (1/8, 12.5%). Six different countries were represented. All of these studies used the TAS-20 for alexithymia assessment [71,120,121,122,123,124,125,126]. Five of the included studies enrolled patients diagnosed with chronic pain [71,120,123,124,125], one study enrolled patients suffering from functional gastrointestinal disorders [126], one study enrolled patients diagnosed with somatization disorder [122], and one study enrolled patients with multisomatoform disorders [121]. Sample sizes ranged from 30 [120] to 154 participants [125]. One study did not report on gender distribution of enrolled participants [120]. One study only included women [123].

#### 3.1.2. Study Descriptions

Aboussouan et al. [123] conducted a non-randomized longitudinal study to examine treatment outcomes in women suffering from chronic pelvic pain (CPP) after a 3–4 week Interdisciplinary Chronic Pain Rehabilitation Program (ICPRP), which included medication management, psychotherapy (individual, family and group), psychoeducation, physical and occupational therapy, weaning from medications and optional monthly aftercare. Fifty-eight women with CPP were age-matched with 58 women with non-pelvic chronic pain (NPCP) as controls. Primary outcomes were operationalized by pain severity (NRS-11; Numeric Rating Scale), depressivity (DASS-21; Depression, Anxiety, and Stress Scale), alexithymia (TAS-20) and impairment in sexual functioning (PDI; Sexual Behavior subscale of Pain Disability Index). The authors conclude that generalized interdisciplinary pain management programs are beneficial for CPP patients and NPCP patients by improvements in impairment in sexual functioning, depressivity and alexithymia. Even though changes within depressivity, alexithymia, and pain were independently associated with improvements in sexual functioning in CPP patients, the authors point out that further research is needed to reveal relationships between these comorbid conditions and sexual functioning within CPP patients. Thereby, they ask for further longitudinal research assessing more time points.

Melin et al. [71] conducted a non-randomized longitudinal study to examine treatment outcomes in patients suffering from chronic benign pain after an Affect School consisting of eight weekly group therapy interventions—with a special theme for every session—and 10 individual sessions of Script Analysis (SA) afterwards to investigate benefits and harms caused by this treatment program. Group sessions focused on recall of occasions when specific affects occurred and how this affect was sensed in mind and body. Fifty-nine patients participated within this study. Primary outcomes were operationalized by alexithymia (TAS-20), anxiousness (HADS-A), depressivity (HADS-D), pain severity (VAS; Visual Analogue Scale), health-related quality of life (EQoL; European Quality of Life health barometer), and stress symptoms (SCI-93; Stress and Crisis Inventory-93). Thirty-six percent of participants matched criteria for alexithymia (TAS-20 ≤ 61). Due to the conducted treatment, which focused on identifying and describing affects and emotions, the authors explain the improvements within DIF and DDF and discuss EOT as a more trait-prone factor. Non-improvements within pain severity were attributed to (a) assessment, (b) emotional, and (c) neurological factors.

Saariaho et al. [124] conducted a non-randomized longitudinal study to examine changes in alexithymia, depressivity, pain intensity and pain disability in patients suffering from chronic non-malignant pain for at least three months. The authors assessed primary outcomes at baseline and at eight-year follow-up. They did not conduct their own treatment approaches. Undergone treatment interventions were assessed retrospectively according to patient’s reports of undergone treatments which were based on a biomedical concept. Out of the initially assessed 271 patients, 83 patients participated at both measurement timepoints. Outcomes were operationalized as alexithymia (TAS-20), depressivity (BDI-II; Beck’s Depression Inventory), pain intensity (VAS; Visual Analogue Scale) and pain disability (PDS; Pain Disability Scale). The authors showed that alexithymia was associated with lessened improvements in pain intensity and disability. They further pointed out that alexithymia and male gender were associated with poorer outcomes after treatment in a sample of patients suffering from chronic pain. Alexithymic patients scored higher on pain disability and depressivity at baseline. At follow-up alexithymic patients scored higher on pain intensity, pain disability, and depressivity. Significant decreases were found in pain intensity, pain disability and depressivity in result of assessed treatment protocols, but they did not find any changes within alexithymia.

Saariaho et al. [125] conducted a non-randomized longitudinal study to examine the effects of alexithymia, depressivity, pain and treatment options in patients suffering from chronic non-malignant pain for at least three months. Primary outcomes were assessed at baseline (T1) and one-year follow-up (T2). Patients did not perform a specific study-based treatment. Instead, the authors assessed the patient records to see if patients had undergone any interventions which were based on a biomedical concept. Out of the initially assessed 271 patients, 154 patients participated at both measurement timepoints. Primary outcomes were alexithymia (TAS-20), depressivity (BDI-II), pain intensity (VAS), and pain disability scores (PDS). Alexithymic patients scored higher on pain disability and depressivity at baseline and follow-up. Significant decreases were found in pain intensity and pain disability in result of assessed treatment protocols. Alexithymia (TAS-20, DIF, and DDF) decreased significantly within the whole sample. In conclusion baseline alexithymia TAS-20 score was identified as significant predictor for pain disability.

Porcelli et al. [126] conducted a non-randomized longitudinal study to examine whether alexithymia was a predictor of treatment outcome in patients suffering from functional gastrointestinal disorders (FGID). They assessed primary outcomes at baseline and at six-month follow-up. Treatments were conducted according to the patient’s symptoms and consisted of combinations of gastrointestinal medications, diet modifications, psychotropic medications, and psychological counseling or brief psychotherapy. Out of the initially assessed 130 patients, 112 patients participated at both measurement timepoints. At baseline, 56% of the whole sample were categorized as alexithymic (TAS-20 ≥ 61). Primary outcomes were gastrointestinal symptoms (GSRS; Gastrointestinal Symptom Rating Scale), alexithymia (TAS-20), anxiousness (HADS-A), and depressivity (HADS-D). On the basis of (a) change within overall gastrointestinal symptoms and (b) low levels of gastrointestinal symptoms at follow-up, the whole sample was divided into “improved” and “unimproved” subgroups. The authors identified alexithymia as the most powerful predictor for recovery status and overall reduction in GSRS symptoms after treatment, even after controlling for baseline GSRS, depressivity, and anxiousness. The TAS-20 total score was shown to be a reliable and stable predictor of treatment outcome. 

Reese [122] conducted a randomized, wait-list-controlled longitudinal study to examine the effects of an individual 10-session CBT, focusing on reduction of physiological arousal through relaxation techniques, enhancing activity regulation, pace activities, increasing awareness of emotions, modifying dysfunctional beliefs, enhancing communication of thoughts and emotions and reducing spousal reinforcement of illness behavior, on alexithymia in patients suffering from somatization disorders. Another aim of this study was to examine whether improvements of alexithymia after CBT predicted improvements in somatization symptoms and functioning. 

Primary outcomes were defined as severity of somatization, measured by Clinical Global Impression for Somatization Disorder (CGI-SD) as well as daily symptom diaries to record maximum severity of somatoform symptoms every day. Other assessed outcomes were defined as mental health (SF-36), physical functioning (Mental Health scale of MOS 26-Item Short-Form Health Survey, SF-36), alexithymia (TAS-20), defensiveness (MCS; Marlowe-Crowne Social Desirability Scale), and somatosensory amplification (SSAS; Somatosensory Amplification Scale). Outcomes were assessed at baseline (T1), 3-months-follow-up (T2), 9-months-follow-up (T3), and 15-months-follow-up (T4). 

Participants not randomized into CBT in combination with psychiatric consultation letter (PCL) treatment group (*n* = 43) were randomized into the control group whose physicians only received a psychiatric consultation letter (PCL; *n* = 41). Out of 367 patients who completed a telephone screening interview, 142 patients participated in a face-to-face screening interview. Finally, 84 patients participated. 

Overall, results revealed a decrease in medically unexplained physical symptoms. A decrease in alexithymia further correlated with improvements in the severity of somatization symptoms. Thus, the authors concluded that CBT successfully improves alexithymia in patients suffering from somatization disorders. Reese [122] attributes these results to a possible shift away from externally oriented thinking towards an awareness of inner feelings and thoughts. He argues that EOT can be seen as ‘the cognitive component’ of alexithymia, whereas DIF and DDF represent ‘the emotional components’. 

Probst et al. [121] conducted a randomized controlled longitudinal study to examine treatment outcomes in patients suffering from multisomatoform disorders after a 12-week manualized brief psychodynamic–interpersonal therapy (PIT) which included establishment of a therapeutic alliance, treatment of somatoform symptoms, behavioral, emotional, and interpersonal correlates, and termination issues. One hundred and seven patients were randomized to treatment condition, and 104 were allocated to the control condition. Out of 107 patients within the treatment condition, 83 remained for statistical analyses due to missing data. Primary outcome was defined as patient-reported physical quality of life at 9-month follow-up after treatment was undertaken and was operationalized by the Physical Component Summary of the SF-36 Health Survey. They did not find significant interactions between the patients′ alliance ratings and alexithymia on physical quality of life at follow-up. Interaction between the therapists′ alliance ratings and the patients′ alexithymia scoring on physical quality of life was significant, but after controlling for depressivity this interaction did not reach significance anymore.

Saedi et al. [120] conducted a randomized longitudinally study to evaluate the efficacy of behavioral–cognitive therapy (CBT) on alexithymia and self-effectiveness of pain in patients suffering from chronic musculoskeletal pain. Out of 45 patients, 30 patients were simple-randomized into two treatment intervention groups and 15 patients to one control group. Treatment consisted of eight sessions of 90 min CBT. Primary outcomes were defined as alexithymia (TAS-20) and self-effectiveness of pain (PSEQ; Pain self-effectiveness questionnaire). Patients underwent CBT decreased significantly more in alexithymia and pain self-efficacy than patients within control group. CBT led to increased self-efficacy of pain, reduced alexithymia, and reduced harmful effects of pain. The authors conclude by pointing out CBT as a beneficial treatment for decreasing alexithymia and increasing the self-effectiveness of pain in patients suffering from chronic musculoskeletal pain. They ascribe the decrease in alexithymia to (a) therapeutic distinction between physical and emotional sensations, as well as (b) emphasis on the role of bodily sensations in stimulating negative thoughts. 

We identified three main foci within these included studies: (1) analyses examining associations between alexithymia and other psychological variables at baseline, (2) analyses examining alexithymia as an outcome variable itself, and (3) analyses examining associations between alexithymia and non-alexithymia-related treatment outcomes. Table 6 provides an overview of types of analyses performed within the included studies.

#### 3.1.3. Associations between Alexithymia and Other Psychological Variables at Baseline

Melin et al. [71] revealed that participants categorized as alexithymic (TAS-20 ≤ 61) scored significantly higher on depressivity (*M*_non alexithymic_ = 7.1, *SD*_non alexithymic_ = 3.6; *M*_alexithymic_ = 11.2, *SD*_alexithymic_ = 4.4; *p* = 0.003), anxiousness (*M*_non alexithymic_ = 7.9, *SD*_non alexithymic_ = 3.7; *M*_alexithymic_ = 11.3, *SD*_alexithymic_ = 3.2; *p* = 0.006) and stress symptoms (*M*_non alexithymic_ = 55.2, *SD*_non alexithymic_ = 19.1; *M*_alexithymic_ = 74.4, *SD*_alexithymic_ = 20.3; *p* = 0.006) at baseline than participants categorized as non-alexithymic (TAS-20 > 61).

Saariaho et al. [124] showed that alexithymic-categorized patients at baseline reported significantly more pain disability (*M*_alexithymic_ = 20.1, *SD*_alexithymic_ = 3.3; *M*_non alexithymic_ = 15.4, *SD*_non alexithymic_ = 4.5; *p* < 0.001, *d* = 1.19) and depressivity (*M*_alexithymic_ = 26.4, *SD*_alexithymic_ = 11.3; *M*_non alexithymic_ = 11.9, *SD*_non alexithymic_ = 8.5; *p* < 0.001, *d* = 1.45) than non-alexithymic-categorized patients. Additionally, a significant correlation was found between the TAS-20 total score and BDI-II (*r* = 0.612, *p* < 0.001) at baseline.

#### 3.1.4. Examination of Alexithymia as Outcome Variable

Aboussouan et al. [123] reported a significant improvement of alexithymia, measured by TAS-20, over all patients (Δ = 6.17, *SE* = 1.19). They did not find any group differences (CPP vs. NPCP) prior to (CPP: *M*_T1_ = 50.81, *SD*_T1_ = 13.86; NPCP: *M*_T1_ = 48.76, *SD*_T1_ = 12.91; *F* (1, 104) = 0.62, *p* = 0.43, *d* = 0.15) and after (CPP: *M*_T2_ = 45.31, *SD*_T2_ = 12.63; NPCP: *M*_T2_ = 42.31, *SD*_T2_ = 10.59; *F* (1, 104) = 1.75, *p* = 0.19, *d* = 0.26) treatment.

Melin et al. [71] reported improvements of alexithymia following treatment. These improvements of alexithymia, measured by TAS-20, remained significant after Bonferroni correction (*p* = 0.004). Across TAS-20 subscales, significant improvements were revealed for DIF and DDF. Changes within TAS-20 (*R*^2^ = 0.03, *p* > 0.05) as well as in DDF (*R*^2^ = 0.02, *p* > 0.05) were independent from a decrease in depressivity. Conversely, however, depressivity explained the variance within DIF (*R*^2^ = 0.11, *p* < 0.05) significantly.

Saariaho et al. [124] did not find any significant change of alexithymia (TAS-20; *M*_T1_ = 46.6, *SD*_T1_ = 13.1; *M*_T2_ = 46.3, *SD*_T2_ = 12.6; *p* = 0.84, *d* = 0.02) or its subscales DIF (*M*_T1_ = 15.2, *SD*_T1_ = 6.7; *M*_T2_ = 14.6, *SD*_T2_ = 6.6; *p* = 0.20, *d* = 0.09), DDF (*M*_T1_ = 10.9, *SD*_T1_ = 4.6; *M*_T2_ = 11.2, *SD*_T2_ = 4.1; *p* = 0.40, *d* = 0.07), or EOT (*M*_T1_ = 20.5, *SD*_T1_ = 5.2; *M*_T2_ = 20.7, *SD*_T2_ = 5.1; *p* = 0.70, *d* = 0.04). At baseline, males scored significantly higher on the TAS-20 total score (*M*_males_ = 50.5, *SD*_males_ = 11.8; *M*_females_ = 44.3, *SD*_females_ = 13.6; *p* = 0.035) and EOT (*M*_males_ = 22.7, *SD*_males_ = 4.2; *M*_females_ = 18.9, *SD*_females_ = 5.1; *p* = 0.001). At follow-up, males scored higher on the TAS-20 total score (*M*_males_ = 49.6, *SD*_males_ = 11.1; *M*_females_ = 44.0, *SD*_females_ = 13.2; *p* = 0.057), and EOT (*M*_males_ = 22.0, *SD*_males_ = 3.5; *M*_females_ = 19.7, *SD*_females_ = 6.0; *p* = 0.057), but neither reached significance.

In a subsequent study, Saariaho et al. [125] found that the percentage of patients categorized as non-alexithymic decreased significantly (T1: 85.0%; T2: 76.5%; *p* = 0.015). Significant changes within outcome variables of the TAS-20 total score (*M*_T1_ = 47.6, *SD*_T1_ = 12.2; *M*_T2_ = 49.7, *SD*_T2_ = 13.1; *p* = 0.005, *d* = 0.166), DIF (*M*_T1_ = 15.7, *SD*_T1_ = 6.3; *M*_T2_ = 16.7, *SD*_T2_ = 7.0; *p* = 0.017, *d* = 0.150), and DDF (*M*_T1_ = 11.1, *SD*_T1_ = 4.4; *M*_T2_ = 12.1, *SD*_T2_ = 4.3; *p* < 0.001, *d* = 0.230) reached significance, but EOT (*M*_T1_ = 20.7, *SD*_T1_ = 4.6; *M*_T2_ = 20.9, *SD*_T2_ = 4.4; *p* = 0.63, *d* = 0.044) did not. Within subgroups of alexithymic-categorized and non-alexithymic-categorized patients, changes were found within the TAS-20 total score (alexithymic: *M*_T1_ = 68.0, *SD*_T1_ = 6.3; *M*_T2_ = 66.6, *SD*_T2_ = 9.7; *p* = 0.034, *d* = 0.171; non-alexithymic: *M*_T1_ = 43.8, *SD*_T1_ = 8.7; *M*_T2_ = 46.7, *SD*_T2_ = 11.3; *p* = 0.001, *d* = 0.288), DIF (alexithymic: *M*_T1_ = 25.5, *SD*_T1_ = 4.7; *M*_T2_ = 25.3, *SD*_T2_ = 5.5; *p* > 0.9, *d* = 0.039; non-alexithymic: *M*_T1_ = 13.9, *SD*_T1_ = 4.7; *M*_T2_ = 15.2, *SD*_T2_ = 6.1; *p* < 0.010, *d* = 0.239), DDF (alexithymic: *M*_T1_ = 17.6, *SD*_T1_ = 3.3; *M*_T2_ = 16.9, *SD*_T2_ = 3.9; *p* = 0.28, *d* = 0.194; non-alexithymic: *M*_T1_ = 10.0, *SD*_T1_ = 3.5; *M*_T2_ = 11.3, *SD*_T2_ = 3.8; *p* < 0.001, *d* = 0.356), and EOT (alexithymic: *M*_T1_ = 24.9, *SD*_T1_ = 3.5; *M*_T2_ = 24.3, *SD*_T2_ = 2.7; *p* = 0.22, *d* = 0.192; non-alexithymic: *M*_T1_ = 20.0, *SD*_T1_ = 4.4; *M*_T2_ = 20.3, *SD*_T2_ = 4.4; *p* = 0.35, *d* = 0.068).

Relative stability of alexithymia was revealed by Porcelli et al. [126] through test–retest reliabilities and resulted in a reliability coefficient of *r* = 0.76, *p* < 0.001. Within the whole sample, the TAS-20 total score (*M*_T1_ = 58.89, *SD*_T1_ = 13.50; *M*_T2_ = 55.85, *SD*_T2_ = 12.32; *t* (222) = 3.56, *p* < 0.01, *d* = 0.68) decreased significantly. The authors showed that, at baseline, unimproved patients scored significantly higher on the TAS-20 total score (*t* (111) = 7.89, *p* < 0.001). Analyses revealed that improved (*M*_T1_ = 52.19, *SD*_T1_ = 12.10; *M*_T2_ = 48.66, *SD*_T2_ = 8.91; *t* (134) = 2.89, *p* < 0.01, *d* = 0.71) and unimproved patients (*M*_T1_ = 69.25, *SD*_T1_ = 7.81; *M*_T2_ = 66.95, *SD*_T2_ = 7.75; *t* (86) = 2.10, *p* < 0.01, *d* = 0.71) did significantly improve within the TAS-20 total score.

Reese [122] identified 24% of participants as alexithymic (TAS-20 ≥ 61) at T1. Analyses revealed baseline alexithymia as well as its subscales to be highly stable over the period of treatment (*r*_TAS-20_ = 0.76, *p*_TAS-20_ < 0.0001; *r*_DIF_ = 0.70, *p*_DIF_ < 0.0001; *r*_DDF_ = 0.72, *p*_DDF_ < 0.0001; *r*_EOT_ = 0.80, *p*_EOT_ < 0.0001). A significant effect for the interaction between time and condition was found for the TAS-20 total score (*F* (2, 68) = 3.36, *p* = 0.04). There was also found a significant effect of time within the TAS-20 total score (*F* (2, 68) = 3.20, *p* = 0.05). Analyses of TAS-20 subscales revealed a significant interaction between time and condition on DIF (*F* (2, 68) = 4.23, *p* = 0.02), indicating that the CBT + PCL group improved more than the PCL group. No significant interaction was found for DDF, whereas the interaction between time and condition proved to be significant within EOT (*F* (2, 138) = 3.04, *p* = 0.05). Both groups differed significantly on changes from T1 to T2 within the TAS-20 total score (*F* (1, 75) = 5.59, *p* = 0.02), DIF (*F* (1, 75) = 6.19, *p* = 0.02), and EOT (*F* (1, 75) = 4.73, *p* = 0.03). Subtracting the alexithymia scores of T2 from alexithymia scores of T4, generated another change score of alexithymia. Analysis revealed that there were no significant group differences within this change score. Alexithymia change score calculated by subtracting alexithymia scores of T1 from alexithymia scores of T4, indicated a significant difference between both groups within DIF (*F* (1, 69) = 5.41, *p* = 0.02), but not within the TAS-20 total score, DDF or EOT. Analyses of change scores of alexithymia, calculated by subtracting alexithymia scores at T2 from alexithymia scores from T4, revealed that treatment conditions differed significantly on EOT (*F* (1, 81) = 4.08, *p* < 0.05), indicating that PCL controls decreased more than participants within the CBT + PCL group. There were no significant difference on the TAS-20 total score, DIF, or DDF. It was also shown that treatment condition predicted changes within the TAS-20 total score (*F* (1, 75) = 5.59, β = −0.26, *p* = 0.02), DIF (*F* (1, 75) = 6.19, β = −0.28, *p* = 0.02), and EOT (*F* (1, 75) = 4.73, β = −0.24, *p* = 0.03), but not DDF.

Analyses conducted by Saedi et al. [120] revealed that patients who underwent CBT improved significantly more in alexithymia (*F* (1) = 50.48, *p* ≤ 0.001) than patients within the control group. Within the intervention group, alexithymia (*M*_T1_ = 69.67, *SD*_T1_ = 9.65; *M*_T2_ = 58.07, *SD*_T2_ = 8.40; *M*_T3_ = 46.67, *SD*_T3_ = 8.86) decreased. Within the control group, alexithymia (*M*_T1_ = 71.07, *SD*_T1_ = 10.44; *M*_T2_ = 70.53, *SD*_T2_ = 10.54; *M*_T3_ = 66.87, *SD*_T3_ = 12.07) remained relatively stable.

#### 3.1.5. Associations between Alexithymia and Non-Alexithymia-Related Treatment Outcomes

Aboussouan et al. [123] found 20.69% of participants matching criteria for alexithymia (TAS-20 ≤ 61). They did not find significant group differences between CPP patients and NPCP patients in alexithymia at baseline and after treatment. In unadjusted analyses, CPP patients remained significantly more impaired in sexual functioning than NPCP patients (*µ*_CPP_ = 4.41 ± 3.36; *µ*_NPCP_ = 2.98 ± 2.87; *F* = 6.03, 1, *p* = 0.016, *d* = 0.46). Linear mixed models revealed significant main effects for time within outcome improvements after treatment regardless of whether participants suffered from CPP (*p* < 0.01; Δ_impairment in sexual function_ = 3.75, *SE*_impairment in sexual function_ = 0.27; Δ_depressivity_ = 13.86, *SE*_depressivity_ = 1.16; Δ_alexithymia_ = 6.17, *SE*_alexithymia_ = 1.19; Δ_pain_ = 3.45, *SE*_pain_ = 0.23). CPP patients decreased significantly more in depressivity than NPCP patients (*p* < 0.05), but did not in impairment in sexual functioning, alexithymia or pain severity. Hierarchical linear regression within CPP patients revealed that marital status and baseline scores of outcome variables together explained 35% of the variance in post-treatment impairment in sexual functioning (*F* (5, 47) = 3.0, *p* = 0.02). Adding change scores of outcome variables increased explanation of the variance in impairment in sexual functioning by 29% (*F* (3, 40) = 8.79, *p* = 0.00).

As Saariaho et al. [124] did show in their study, pain intensity (*M*_T1_ = 5.7, *SD*_T1_ = 1.2; *M*_T2_ = 4.6, *SD*_T2_ = 2.0; *p* < 0.001, *d* = 0.67), pain disability (*M*_T1_ = 16.3, *SD*_T1_ = 4.7; *M*_T2_ = 11.2, *SD*_T2_ = 6.2; *p* < 0.001, *d* = 0.93), and depressivity (*M*_T1_ = 14.7, *SD*_T1_ = 11.0; *M*_T2_ = 10.8, *SD*_T2_ = 9.1; *p* < 0.001, *d* = 0.36) decreased within the whole sample. At follow up alexithymic subjects reported more pain intensity (*M*_alexithymic_ = 5.6, *SD*_alexithymic_ = 1.2; *M*_non alexithymic_ = 4.4, *SD*_non alexithymic_ = 2.1; *p* = 0.034, *d* = 0.70), pain disability (*M*_alexithymic_ = 15.2, *SD*_alexithymic_ = 6.0; *M*_non alexithymic_ = 11.1, *SD*_non alexithymic_ = 6.0; *p* = 0.015, *d* = 0.068) and depressivity (*M*_alexithymic_ = 20.8, *SD*_alexithymic_ = 11.2; *M*_non alexithymic_ = 8.5, *SD*_non alexithymic_ = 6.8; *p* < 0.001, *d* = 1.33) than non-alexithymic subjects. Male gender (improvers: 5/18; non-improvers: 28/31; *p* = 0.33, *φ* = 0.24), baseline TAS-20 score (*M*_improvers_ = 41.1, *SD*_improvers_ = 8.1; *M*_non-improvers_ = 48.8, *SD*_non-improvers_ = 14.0; *p* = 0.016, *d* = 0.67) and DDF (*M*_improvers_ = 9.2, *SD*_improvers_ = 2.5; *M*_non-improvers_ = 11.6, *SD*_non-improvers_ = 5.0; *p* = 0.034, *d* = 0.61) were significantly higher in the group without improvement within pain intensity. DIF (*M*_improvers_ = 13.0, *SD*_improvers_ = 4.1; *M*_non-improvers_ = 16.2, *SD*_non-improvers_ = 7.3; *p* = 0.052, *d* = 0.54) and EOT (*M*_improvers_ = 18.9, *SD*_improvers_ = 5.0; *M*_non-improvers_ = 21.0, *SD*_non-improvers_ = 5.1; *p* = 0.097, *d* = 0.42) did not differ significantly between improvers and non-improvers within pain intensity. A significant correlation was found between the TAS-20 total score and BDI-II at follow-up (*r* = 0.743, *p* < 0.001). At follow-up males scored higher on pain intensity (*M*_males_ = 5.2, *SD*_males_ = 1.8; *M*_females_ = 4.3, *SD*_females_ = 2.1; *p* = 0.051), and pain disability (*M*_males_ = 13.6, *SD*_males_ = 5.7; *M*_females_ = 10.6, *SD*_females_ = 6.3; *p* = 0.033).

Saariaho et al. [125] showed that over the period of their study the percentage of patients with acceptable pain intensity (VAS ≤ 4; baseline: 7.2%, follow-up: 26.1%, *p* < 0.001) and pain disability (PDS ≤ 13; T1: 28.9%, T2: 44.7%, *p* < 0.001) increased. Over all patients significant changes within outcome variables of pain intensity (*M*_T1_ = 5.9, *SD*_T1_ = 1.3; *M*_T2_ = 5.1, *SD*_T2_ = 1.9; *p* < 0.001, *d* = 0.491) and pain disability (*M*_T1_ = 16.3, *SD*_T1_ = 4.9; *M*_T2_ = 14.3, *SD*_T2_ = 6.0; *p* < 0.001, *d* = 0.365) reached significance, but depressivity (BDI-II; *M*_T1_ = 15.7, *SD*_T1_= 10.8; *M*_T2_ = 15.4, *SD*_T2_ = 11.6; *p* = 0.58, *d* = 0.027) did not. Within subgroups of alexithymic-categorized and non-alexithymic-categorized patients changes were found within pain intensity (alexithymic: *M*_T1_ = 6.3, *SD*_T1_ = 1.4; *M*_T2_ = 5.7, *SD*_T2_ = 1.8; *p* = 0.006, *d* = 0.372; non-alexithymic: *M*_T1_ = 5.9, *SD*_T1_ = 1.3; *M*_T2_ = 4.9, *SD*_T2_ = 1.9; *p* < 0.001, *d* = 0.614), pain disability (alexithymic: *M*_T1_ = 19.1, *SD*_T1_ = 3.2; *M*_T2_ = 17.4, *SD*_T2_ = 5.3; *p* = 0.034, *d* = 0.388; non-alexithymic: *M*_T1_ = 15.8, *SD*_T1_ = 5.0; *M*_T2_ = 13.7, *SD*_T2_ = 6.0; *p* < 0.001, *d* = 0.362), and BDI-II (alexithymic: *M*_T1_ = 27.3, *SD*_T1_ = 9.8; *M*_T2_ = 28.7, *SD*_T2_ = 12.9; *p* = 0.46, *d* = 0.122; non-alexithymic: *M*_T1_ = 13.6, *SD*_T1_ = 9.5; *M*_T2_ = 13.1, *SD*_T2_ = 9.6; *p* = 0.39, *d* = 0.052).

Porcelli et al. [126] found that within the whole sample TAS-20 total score (*M*_T1_ = 58.89, *SD*_T1_ = 13.50; *M*_T2_ = 55.85, *SD*_T2_ = 12.32; *t* (222) = 3.56, *p* < 0.01, *d* = 0.68), anxiousness (*M*_T1_ = 10.15, *SD*_T1_ = 4.98; *M*_T2_ = 7.67, *SD*_T2_ = 5.08; *t* (222) = 6.42, *p* < 0.01, *d* = 1.22), depressivity (*M*_T1_ = 10.63, *SD*_T1_ = 5.68; *M*_T2_ = 8.02, *SD*_T2_ = 5.61; *t* (222) = 5.59, *p* < 0.01, *d* = 1.25), and GSRS total score (*M*_T1_ = 11.00, *SD*_T1_ = 5.18; *M*_T2_ = 5.77, *SD*_T2_ = 6.14; *t* (222) = 12.12, *p* < 0.01, *d* = 2.30) decreased significantly. At baseline, unimproved patients scored significantly higher on anxiousness (*t* (111) = 1.98, *p* = 0.04), depressivity (*t* (111) = 4.39, *p* < 0.001, and GSRS total score (*t* (111) = 3.62, *p* = 0.005). Analyses revealed that improved patients significantly improved on anxiousness (*M*_T1_ = 9.41, *SD*_T1_ = 4.50; *M*_T2_ = 5.53, *SD*_T2_ = 3.65; *t* (134) = 8.52, *p* < 0.01, *d* = 2.08), depressivity (*M*_T1_ = 8.91, *SD*_T1_ = 5.20; *M*_T2_ = 5.18, *SD*_T2_ = 3.91; *t* (134) = 7.37, *p* < 0.01, *d* = 1.80), and GSRS total scores (*M*_T1_ = 9.65, *SD*_T1_ = 4.15; *M*_T2_ = 1.75, *SD*_T2_ = 1.26; *t* (134) = 18.32, *p* < 0.01, *d* = 4.48). Unimproved patients did not improve on anxiousness (*M*_T1_ = 11.30, *SD*_T1_ = 5.49; *M*_T2_ = 10.95, *SD*_T2_ = 5.27; *t* (86) = 0.61, *d* = 0.19), depressivity (*M*_T1_ = 13.30, *SD*_T1_ = 5.40; *M*_T2_ = 12.41, *SD*_T2_ = 4.99; *t* (86) = 1.61, *d* = 0.49), and GSRS total scores (*M*_T1_ = 13.09, *SD*_T1_ = 5.93; *M*_T2_ = 11.98, *SD*_T2_ = 5.45; *t* (86) = 3.12, *d* = 0.95). 

The authors further conducted logistic regressions that specified treatment outcome (improved/unimproved) as dependent variable and baseline TAS-20, anxiousness, depressivity, and GSRS as independent variables. Results revealed the TAS-20 as strongest predictor of treatment outcome (*R*^2^ = 0.38, *X*^2^(1) = 53.64, *p* < 0.01), followed by depressivity (Cox and Snell *R*^2^ = 0.14, *X*^2^(1) = 16.84, *p* < 0.01), GSRS total score (Cox and Snell *R*^2^ = 0.10, *X*^2^(1) = 12.21, *p* < 0.01), and anxiousness (Cox and Snell *R*^2^ = 0.03, *X*^2^(1) = 3.93, *p* < 0.05). Within hierarchical regression analyses the first step including anxiousness, depressivity, and GSRS total score showed a significant fit of the model (*X*^2^(3) = 28.97, *p* < 0.001, Cox and Snell *R*^2^ = 0.23). Adding baseline TAS-20 showed an increase in overall fit of the model (*X*^2^(4) = 61.48, *p* < 0.001, Cox and Snell *R*^2^ = 0.42) and thereby increased prediction of improved patients from 84% to 85% and unimproved patients from 66% to 82%. Conducting a hierarchical regression, adding only TAS-20 as first step, the model revealed significance (*X*^2^(1) = 53.64, *p* < 0.001, Cox and Snell *R*^2^ = 0.38). By adding anxiousness, depressivity and GSRS total scores as second step the fit of the model as still significant (*X*^2^ (1) = 7.84, *p* < 0.05, Cox and Snell *R*^2^ = 0.42). Percentage changes within GSRS were significantly predicted by the TAS-20 total score (*F* (1, 110) = 45.36, *p* < 0.05, *R*^2^ = 0.29), depressivity (*F* (1, 110) = 22.14, *p* < 0.01, *R*^2^ = 0.17), and anxiousness (*F* (1, 110) = 4.93, *p* < 0.05, *R*^2^ = 0.04). Within another conducted hierarchical regression anxiousness and depressivity added as step 1 the model showed significant prediction (*F* (2, 109) = 11.47, *p* < 0.01, *R*^2^ = 0.17) of percentage change of GSRS. By adding TAS-20 to the regression as step 2 a significant increase in incremental variance could be shown (*F* (3, 108) = 18.55, *p* < 0.01, *R*^2^ = 0.34).

Reese [122] showed that improvement in somatization symptoms at T2 correlated with changes within the TAS-20 total score (*r* = 0.33, *p* < 0.01), DIF (*r* = 0.26, *p* < 0.01), and EOT (*r* = 0.34, *p* < 0.01), but not DDF (*r* = 0.11, *p* = n.s.) from T1 to T4. Improvement in somatization symptoms at T4 were associated with changes within the TAS-20 total score (*r* = 0.34, *p* < 0.01), DIF (*r* = 0.32, *p* < 0.01), DDF *r* = 0.27, *p* < 0.05), but not EOT (*r* = 0.13, *p* = n.s.) from T1 to T4. There were also associations between physical functioning at T2 and changes from T1 to T4 within the TAS-20 total score (*r* = −0.26, *p* < 0.05), DIF (*r* = −0.27, *p* < 0.05), DDF (*r* = −0.23, *p* < 0.05), but not EOT (*r* = −0.01, *p* = n.s.) and between physical functioning at T4 and changes from T1 to T4 within the TAS-20 total score (*r* = −0.32, *p* < 0.01), DIF (*r* = −0.37, *p* < 0.01), but not DDF (*r* = −0.20, *p* = n.s.), and EOT (*r* = −0.09, *p* = n.s.). Analyses also revealed correlations between work status and changes in the TAS-20 total score (*r* = 0.26, *p* = 0.02), and DDF (*r* = 0.32, *p* = 0.003) at T2, and changes within EOT between T1 and T2 were associated with the duration of symptoms (*r* = −0.31, *p* = 0.005), meaning that the longer the symptoms lasted, the more EOT decreased. Analyses comparing both groups revealed that the CBT + PCL group improved more in somatization symptoms than the PCL group from T1 to T2 (*F* (1, 82) = 36.06, *p* < 0.0001) and from T1 to T4 (*F* (1, 82) = 27.85, *p* < 0.0001). Analyses focusing on secondary outcomes revealed a significant interaction between time and condition in diary scores, meaning that the CBT + PCL group decreased more in diary scores of severity of somatoform symptoms (*F* (2, 69) = 5.52, *p* = 0.005). Another significant interaction between time and condition was found in physical functioning (*F* (2, 69) = 5.39, *p* = 0.007), meaning that the CBT + PCL group improved more than the PCL group. It was also shown that patients identified as alexithymic (TAS-20 ≥ 61) scored lower on mental health than patients defined as non-alexithymic (TAS-20 ≤ 51) did at T1 (*F* (1, 67) = 9.74, *p* = 0.003); this difference was no longer significant at T2 and T4. Patients identified as alexithymic scored higher on anxiousness than patients identified as non-alexithymic at T1 (*F* (1, 67) = 14.73, *p* = 0.0003) and T4 (*F* (1, 59) = 5.07, *p* = 0.02). Groups differed significantly on physical functioning, when controlling for physical functioning at T1, meaning that patients identified as alexithymic reported greater physical functioning at T4 (*F* (1, 62) = 10.29, *p* < 0.002). Alexithymic patients scored lower on defensiveness at T1 (*F* (1, 67) = 4.04, *p* < 0.05). There were no significant differences between groups within CGI-SD at T1, on improvement in CGI-SD, and somatosensory amplification. The CBT + PCL group improved more in somatization symptoms from T1 to T2 (*F* (1, 82) = 30.51, *p* < 0.0001), and at T4 (*F* (1, 82) = 27.85, *p* < 0.0001).

Mediational analyses revealed treatment condition as significant predictor for improvements within somatization symptoms at T2 (*F* (1, 75) = 36.06, β = −0.57, *p* < 0.0001) and T4 (*F* (1, 70) = 28.89, β = −0.54, *p* < 0.0001). Improvements within somatization symptoms at T2 were predicted by changes in the TAS-20 total score (*F* (1, 75) = 9.25, β = 0.33, *p* = 0.003), DIF (*F* (1, 75) = 5.61, β = 0.26, *p* = 0.02), and EOT (*F* (1, 75) = 9.68, β = 0.34, *p* = 0.003) and improvements in somatization symptoms at T4 were predicted by the TAS-20 total score (*F* (1, 70) = 9.06, β = 0.34, *p* = 0.004), DIF (*F* (1, 70) = 8.21, β = 0.34, *p* = 0.006), and DDF (*F* (1, 70) = 5.33, β = 0.27, *p* = 0.02). Model including treatment condition and the TAS-20 total score as predictors for improvements in somatization symptoms at T2, reached significance (*F* (2, 74) = 20.81, β = 0.19, *p* < 0.05). In final model changes within the TAS-20 total score was no longer a significant predictor for improvement in somatization symptoms when controlling for mental health, at T2, severity of somatization at T1, and defensiveness at T2, and somatosensory amplification. Final model reached significance (*F* (5, 71) = 9.39, *p* < 0.0001) and accounted for 40% of the variance in improvement in somatization symptoms at T2. Within final model just treatment condition proved to be a significant predictor (β = −0.49, *p* < 0.0001). The interaction between treatment condition and changes within the TAS-20 total score significantly predicted improvement in somatization symptoms at T2 (*F* (1, 75) = 5.48, *p* = 0.02) and accounted for 7% of the variance but was no longer significant when condition and treatment added separately to the model (*p* = 0.32). Interaction between condition and changes within EOT reached significance when entered alone into the regression model (*F* (1, 75) = 8.38, *p* = 0.005) and accounted for 10% of the variance in somatization symptoms at T2. Interactions between condition and DIF or DDF did not reach significance. Even though changes within DDF significantly predicted improvement in somatization symptoms at T4, DDF cannot seen as a mediator, because treatment condition did not predict changes within DDF. The TAS-20 total score and DIF did not reach significance as predictors for improvements in somatization symptoms at T4.

Mediational analyses focusing on physical functioning as outcome revealed treatment condition as a significant predictor for physical functioning at T2 (*F* (2, 74) = 158.53, β = 0.16, *p* = 0.01) and T4 (*F* (2, 69) = 48.92, β = 0.20, *p* = 0.01) when controlling for physical functioning at T1. Physical functioning at T2 was predicted by changes within the TAS-20 total score and changes within DIF when controlling for physical functioning at T1, defensiveness at T2, mental health at T2, and somatosensory amplification at T2. Mediational status of the TAS-20 total (*z* = 1.40, *p* = 0.16) score and DIF (*z* = 1.41, *p* = 0.16) for physical functioning at T2 did not reach significance. Physical functioning at T4 was significantly predicted by changes within the TAS-20 total score (*F* (2, 69) = 50.84, β = −0.23, *p* = 0.01) and changes within DIF (*F* (2, 69) = 52.79, β = −0.25, *p* = 0.002) when controlling for physical functioning at T1. Within the final model, physical functioning at T4 was significantly predicted by physical functioning at T1 (*F* (6, 58) = 15.62, β = 0.69, *p* < 0.0001) and changes within the TAS-20 total score (*F* (6, 58) = 15.62, β = −0.21, *p* < 0.05).

Mediational analyses focusing on daily symptom diary scores as outcome revealed treatment condition as a significant predictor of daily symptom diary scores at T2 (*F* (2, 74) = 11.45, β = −0.29, *p* = 0.006) and T4 (*F* (2, 69) = 17.53, β = −0.31, *p* = 0.002) when controlling for daily symptom diary scores at T1. EOT significantly predicted daily symptom diary scores at T2 when controlling for daily symptom diary scores at T1 (β = 0.26, *p* = 0.02) and proved to be a significant predictor for daily symptom diary scores at T2 when controlling for daily symptom diary scores at T1, defensiveness at T2, mental health at T2, and somatosensory amplification at T2 (β = 0.30, *p* = 0.01), but the Sobel test did not reach significance (*z* = −1.74, *p* = 0.08). For daily symptom diary scores at T4, no variable proved to be a significant predictor.

Analyses by Probst et al. [121] revealed a significant association between TAS-20 total scores and PHQ-9 scores (*r* = 0.414, *p* < 0.01). Moderation analyses investigating alexithymia as a moderator of the association between the patient’s alliance ratings and physical quality of life revealed a non-significant interaction effect (*t* = 0.58, *p* = 0.57), but alexithymia was identified as a significant moderator for the association between the therapist’s alliance ratings and physical quality of life (*t* = 2.01, *p* < 0.05). The moderating value defining the Johnsons–Neyman significance region was shown to be a TAS-20 total score of 61.21. Adding baseline depressivity as covariate, the association between the therapist’s alliance and physical quality of life after treatment no longer reached significance (*t* = 1.83, *p* = 0.07).

Saedi et al. [120] revealed that patients who underwent CBT improved significantly more in self-effectiveness of pain (*F* (1) = 89.89, *p* ≤ 0.001). Self-effectiveness of pain (*M*_T1_ = 17.60, *SD*_T1_ = 3.90; *M*_T2_ = 25.13, *SD*_T2_ = 4.03; *M*_T3_ = 35.93, *SD*_T3_ = 6.0) increased over time. Within the control group, the self-efficacy of pain (*M*_T1_ = 17.47, *SD*_T1_ = 1.80; *M*_T2_ = 16.73, *SD*_T2_ = 2.40; *M*_T3_ = 16.40, *SD*_T3_ = 1.92) remained relatively stable.

#### 3.1.6. Risk of Bias

Table 7 gives an overview of the results of risk of bias assessment for the included RCTs. The complete assessment can be found in Supplementary Table S3. Table 8 gives an overview of the results of risk of bias assessment for included NRCTs. The complete assessment can be found in Supplementary Table S4.

### 3.2. Alexithymia and Tinnitus

#### 3.2.1. Study Characteristics

For the second part of this systematic review, three studies met the inclusion criteria (see Table 9). Three different countries are represented within this review. Two of the included studies were conducted in the European Region (2/3, 66.6%) and one within the South-East Asia Region (1/3, 33.3%). All of these studies were designed as cross-sectional studies and assessed alexithymia via TAS-20 [107,108,109]. Tinnitus was assessed by THI by Bakhla et al. [107] and Wielopolskiet al. [108]. Salonen et al. [109] used an individual tinnitus questionnaire. Sample sizes ranged from 70 [107] to 583 subjects [109].

#### 3.2.2. Study Descriptions

Bakhla et al. [107] conducted a non-randomized cross-sectional study to examine the prevalence and associations of alexithymia, depressivity, and anxiousness in patients suffering from both unilateral or bilateral tinnitus. To reach this aim, they assessed TAS-20, THI, HADS-A and HADS-D within 70 clinical patients.

Salonen et al. [109] conducted a non-randomized cross-sectional study to evaluate associations between tinnitus, depressivity, and alexithymia in elderly patients. Five hundred and eighty-three patients, aged between 70 and 85 years, comprised the study sample. For psychometric assessment, TAS-20, BDI, and an individual “questionnaire on the presence or absence of tinnitus, the degree of annoyance caused by tinnitus, the audibility of tinnitus and the nature of the sound” were used. Patients also received audiometric measurements to identify impaired hearing.

Wielopolski et al. [108] conducted a non-randomized cross-sectional study to evaluate which alexithymic characteristics are linked to the subjective experience of tinnitus. Therefore, the study sample was comprised of 207 patients suffering from tinnitus for at least one month. For psychometric assessment, TAS-20, BDI, and THI were used.

We identified three main foci within these included studies: (1) analyses examining prevalence rates of alexithymia in patients with chronic tinnitus, (2) analyses examining associations between alexithymia and tinnitus-related distress, and (3) analyses examining differences in alexithymia in patients with bothersome versus non-bothersome tinnitus. Table 10 gives an overview of types of analyses performed within included studies.

#### 3.2.3. Prevalence Rates of Alexithymia (TAS-20 ≥ 61) in Patients with Chronic Tinnitus

Analyses conducted by Bakhla et al. [107] showed that within the whole sample, tinnitus-related distress measured by THI was mostly severe (34.3%), moderate (20.0%), catastrophic (18.6%), mild (17.1%), and slight (10.0%). Point prevalence of alexithymia (TAS-20 ≥ 61) within this sample was found to be 65.71%.

Within the sample of Wielopolski et al. [108], tinnitus-related distress measured by THI was distributed as following: slight (12.6%), mild (29.0%), moderate (28.0%), severe (21.7%), and catastrophic (8.7%). Point prevalence of alexithymia (TAS-20 ≥ 61) was found to be 9.2%.

#### 3.2.4. Associations between Alexithymia and Tinnitus-Related Distress

Bakhla et al. [107] divided the whole sample due to statistics of THI (*M* = 53.92, *SD* = 2.46) into two groups: (1) highly bothered by tinnitus (THI ≥ 54), and (2) less bothered by tinnitus (THI ≤ 54). They reported that patients categorized as highly bothered by tinnitus (THI ≥ 54) scored significantly higher on alexithymia measured by the TAS-20 total score (*M*_highly bothered_ = 68.34, *SD*_highly bothered_ = 8.73; *M*_low bothered_ = 58.22, *SD*_low bothered_ = 11.22; *U*(*N*_highly bothered_ = 43, *N*_low bothered_ = 27) = 274.50, *z* = −3.695, *p* = 0.000), DIF (*M*_highly bothered_ = 24.16, *SD*_highly bothered_ = 4.97; *M*_low bothered_ = 17.07, *SD*_low bothered_ = 5.21; *U*(*N*_highly bothered_ = 43, *N*_low bothered_ = 27) = 197.00, *z* = −4.636, *p* = 0.000), and DDF (*M*_highly bothered_ = 17.37, *SD*_highly bothered_ = 3.50; *M*_low bothered_ = 14.18, *SD*_low bothered_ = 4.30; *U*(*N*_highly bothered_ = 43, *N*_low bothered_ = 27) = 316.00, *z* = −3.212, *p* = 0.001), but not on EOT (*M*_highly bothered_ = 26.81, *SD*_highly bothered_ = 3.26; *M*_low bothered_ = 26.96, *SD*_low bothered_ = 4.34; *U*(*N*_highly bothered_ = 43, *N*_low bothered_ = 27) = 553.50, *z* = −0.327, *p* = 0.743). Highly bothered patients also scored significantly higher on depressivity measured by HADS-D (*M*_highly bothered_ = 9.06, *SD*_highly bothered_ = 3.46; *M*_low bothered_ = 4.33, *SD*_low bothered_ = 2.96; *U*(*N*_highly bothered_ = 43, *N*_low bothered_ = 27) = 168.00, *z* = −4.994, *p* = 0.000) and anxiousness measured by HADS-A (*M*_highly bothered_ = 10.74, *SD*_highly bothered_ = 4.01; *M*_low bothered_ = 5.81, *SD*_low bothered_ = 3.92; *U*(*N*_highly bothered_ = 43, *N*_low bothered_ = 27) = 214.00, *z* = −4.433, *p* = 0.000). These findings suggest DIF and DDF are more likely to be associated with tinnitus-related distress than EOT. This study also revealed a high association between tinnitus and (a) alexithymia, (b) anxiousness, and (c) depressivity. The authors ask for further research with bigger sample sizes and assessment via structured psychiatric interviews.

Wielopolski et al. [108] found a mean of TAS-20 of 44.0 (*SD* = 10.8). Conducted correlational analyses revealed a significant association between the TAS-20 total score and THI total score (*r* = 0.33, *p* = < 0.01), TAS-20 total score and THI functional (*r* = 0.33, *p* < 0.01), TAS-20 total score and THI emotional (*r* = 0.29, *p* < 0.01), and TAS-20 total score and THI catastrophic (*r* = 0.30, *p* < 0.01). THI total score was also significantly associated with the TAS-20 subscales DIF (*r* = 0.46, *p* < 0.01), DDF (*r* = 0.28, *p* < 0.01), but not EOT (*r* = 0.02, *p* = n.s.). THI total score was also highly associated with BDI score (*r* = 0.70, *p* < 0.01). The TAS-20 subscale DIF was additionally found to be associated with THI functional (*r* = 0.45, *p* < 0.01), THI emotional (*r* = 0.42, *p* < 0.01), and THI catastrophic (*r* = 0.41, *p* < 0.01). DDF was associated with THI functional (*r* = 0.28, *p* < 0.01), THI emotional (*r* = 0.26, *p* < 0.01), and THI catastrophic (*r* = 0.26, *p* < 0.01). EOT did not associate significantly with THI total score and its subscales. Within hierarchical regression analyses, BDI (β = 0.64, *R*^2^_adjusted_ = 0.49, *p* < 0.01) and DIF (β = 0.12, *R*^2^_adjusted_ = 0.50, *p* < 0.05) predicted tinnitus-related distress significantly. DIF was significantly predicted by THI functional (β = 0.45, *R*^2^_adjusted_ = 0.20, *p* < 0.01). The authors interpreted their findings as a moderate association between the subjective tinnitus-related distress and alexithymia. They ask to widen the focus of research, as they point out that not just alexithymia but also more general impairments in awareness and regulation of mental states may cause differences within subjective tinnitus-related distress.

#### 3.2.5. Differences in Alexithymia in Patients with Bothersome versus Non-Bothersome Tinnitus

Salonen et al. [109] divided the whole sample due to the used individual tinnitus questionnaire into three subgroups: [1] tinnitus with annoyance, [2] tinnitus without annoyance, and [3] no tinnitus. Statistics revealed that the biggest subgroup was built by patients without tinnitus (*N* = 228, *n*_female_ = 146, *n*_male_ = 82), followed by tinnitus without annoyance (*N* = 180, *n*_female_ = 102, *n*_male_ = 78), and tinnitus with annoyance (*N* = 163, *n*_female_ = 89, *n*_male_ = 74). Within the whole sample, 123 patients were identified as alexithymic (TAS-20 ≥ 61). Point prevalence of alexithymia was found to be 14.9% in patients without tinnitus, 27.8% in patients suffering from tinnitus without annoyance, and 23.3% in patients suffering from tinnitus with annoyance. Analyses revealed similar results for association between the TAS-20 total score and tinnitus: patients suffering from tinnitus without annoyance were found to score the highest on alexithymia (*M* = 53.6, *SD* = 10.8), followed by patients suffering from tinnitus with annoyance (*M* = 52.1, *SD* = 11.6), and participants without tinnitus (*M* = 49.9, *SD* = 10.3). These groups differed significantly (*p* = 0.002, *p_adjusted_* = 0.010). Patients with impaired hearing were more commonly alexithymic (23.6%) than patients without impaired hearing (14.3%), *p* = 0.012. Patients with impaired hearing scored significantly higher on the TAS-20 total score than patients without impaired hearing (*M_impaired hearing_* = 52.4, *SD_impaired hearing_* = 10.7; *M*_not impaired hearing_ = 49.6, *SD*_not impaired hearing_ = 11.2, *p* = 0.006); this association did not remain significant when adjusting for multivariate analysis adding sociodemographics as control variables. The authors found an association between alexithymia and tinnitus in elderly people, but detailed analyses showed that alexithymia is not helpful in explaining tinnitus annoyance within this elderly sample. They ask for further research on this topic within a population-based study or a case-controlled study examining patients who are seriously suffering from tinnitus.

#### 3.2.6. Risk of Bias

The assessments of risk of bias for the three included studies were carried out by the JBI Critical Appraisal Checklist for Analytical Cross Sectional Studies [130]. All of the included studies are at just a low risk of bias, as mentioned in Table 11.

## 4. Discussion

This systematic review was divided into two individual parts: 

First, it aimed to collate the current evidence of (1) whether, and if so, (2) to what extent alexithymia affects therapeutic outcomes in patients with somatoform symptom presentations. Due to an already published review focusing on associations between alexithymia and somatoform presentations by De Gucht et al. [37], we only included studies published after 2001. 

Second, the review aimed to collate findings on alexithymia in patients with chronic tinnitus—which may conceptually overlap with somatoform symptom presentations. We summarized the key findings of each identified study according to our review foci. Each summary highlights the aims, methods, results, and unanswered questions of the particular study.

### 4.1. Alexithymia and Somatoform Conditions

#### 4.1.1. Summary of Main Results

The majority of the included studies reported significant improvement of alexithymia in patients with somatoform conditions following psychotherapeutic intervention programs [71,120,122,123,126].

Of the studies that report significant TAS-20 improvements, only two studies also analyzed the TAS-20 subscales. Melin et al. [71] report significant improvements in DIF and DDF, whereas Reese [122] reports significant improvements in DIF and EOT. These findings are in keeping with the results of other studies that investigated changes of alexithymia in cohorts suffering from other psychological conditions [131,132,133].

Two included studies did not conduct interventions, but asked participants about any type of treatment they had undergone between study admission and follow-up measurement [124,125]. In their 1-year follow-up study, Saariaho et al. [125] report increased alexithymia (operationalized via higher scores in the TAS-20 total, DIF and DDF scores). Investigating alexithymia in their 8-year follow-up study, no changes of alexithymia were found [124]. This finding may represent the assumed stability of alexithymia when not directly addressed by therapeutic interventions [134,135,136].

All conducted treatment programs improved the somatoform conditions that patients presented with at baseline. Improvements were found for sexual functioning [123], chronic pain [123,124,125], somatization symptoms [122], and gastrointestinal symptoms [126]. Conducted treatments also affected and improved conditions like depressivity [71,123,124,126], anxiousness [126], quality of life [71], and physical functioning [122].

In addition, TAS-20 scores correlated with somatoform symptom clusters. Higher baseline TAS-20 total scores predicted significantly higher baseline depressivity, anxiousness and stress symptoms [71] as well as pain disability [125]. TAS-20 total scores correlated significantly with functional gastrointestinal symptoms [126], depressivity [121] and anxiousness [122] at baseline and follow-up. Prior to treatment, alexithymic (TAS-20 ≥ 61), compared to non-alexithymic patients (TAS-20 < 61), reported significantly more pain disability and depressivity [124,125], and significantly more pain intensity, pain disability and depressivity at follow-up [124]. Subsequently, Saariaho et al. [125] identified the TAS-20 total score as a significant predictor for pain disability at follow-up. Subjects categorized as alexithymic (TAS-20 ≥ 61) were also found to score higher on anxiousness at baseline and after treatment than non-alexithymic subjects [122]. Another main finding was that mental health was negatively correlated with the TAS-20 total score and all its subscales at baseline, meaning the higher the TAS-20 scores, the poorer the mental health [121,122]. This finding is in line with Quinto et al. [137], who also report on this negative correlation.

Comparison of the included studies is difficult due to different considered somatoform conditions (and the broadness of the somatization spectrum), different types of conducted psychotherapeutic interventions, and a possible location bias.

#### 4.1.2. Location Bias

Using electronic databases as well as searches within reference lists for identification of relevant studies focusing on alexithymia in the context of somatoform conditions, we included eight studies from six different countries. The majority of the included studies were conducted in the European Region (*n* = 5), followed by the Region of the Americas (*n* = 2), and the Eastern Mediterranean Region (*n* = 1). No included study was conducted in the African Region, the South-East Asia Region, or the Western Pacific Region. Given that the majority of included studies were conducted in developed countries, the possibility of location bias must be acknowledged. To some extent, this location bias might be explained by language restrictions that we defined for study inclusion. We restricted our search to studies published in English or German. Jüni et al. [138] revealed that non-English studies are typically complex to identify and that the need for inclusion of non-English studies depends on the particular topic of the conducted review. If the content area of a review is primarily in the published literature, a review based on a search for studies in English is likely to produce similar results to a review based on studies without language restrictions [139].

#### 4.1.3. Conclusions

In accordance with Rudolf and Henningsen [140] and Gottschalk and Rief [141], psychological and psychosomatic treatment approaches constitute the gold-standard treatment for somatoform symptom presentations and related psychological conditions. 

Rudolf and Henningsen [140] further emphasize the relevance of therapeutically addressing interpersonal patterns that commonly maintain somatoform difficulties. Spitzer et al. [142] showed that high scores of alexithymia are more likely associated with a cold and socially-avoiding interpersonal behavior. They conclude by proposing “that alexithymia involves not only an impaired ability to regulate emotions internally, but also represents a reduced capacity to use social interactions for affect regulation” [142].

The summarized results demonstrate that alexithymia is a relevant variable in patients with somatoform conditions. Psychotherapeutic interventions lead to improvement of alexithymia and somatoform conditions as well as related psychological constructs like depressivity or anxiousness. Treatment conceptualizations for patients with alexithymic characteristics may focus on improving patients’ attentional control over interoceptive signals [143] or use emotion-focused treatment strategies to facilitate emotional awareness and, thereby improve both alexithymia and somatization phenomena.

### 4.2. Alexithymia and Chronic Tinnitus

#### 4.2.1. Summary of Main Results

Prevalence of alexithymia in patients suffering from chronic tinnitus was found to be 65.7% [107]. Salonen et al. [109] revealed a significant association between the expression of alexithymia and the presence of tinnitus.

Wielopolski et al. [108] identified DIF and BDI as significant predictors of tinnitus-related distress. Patients categorized as highly bothered by tinnitus (THI ≥ 54) scored significantly higher on depressivity and anxiousness as well as on the TAS-20, DIF and DDF but not EOT scores [107]. The authors further found that the THI subscales—functional, emotional and catastrophic—were significantly associated with the TAS-20, DIF and DDF but not EOT scores. Follow-up analyses revealed that the functional subscale of the THI was specifically associated with higher DIF scores [108]. Analyzing all patients together, Salonen et al. [109] showed an association between TAS-20 and the presence of tinnitus. After Salonen et al. [109] grouped the whole sample into “annoyed” vs. “not annoyed” by their tinnitus, no significant associations between TAS-20 and tinnitus-related distress scores were found. Contrary to expectations, significant differences emerged between “not annoyed” patients with chronic tinnitus who showed higher TAS-20 scores than “annoyed” patients with chronic tinnitus who, in turn, showed higher TAS-20 scores than non-patient controls [109].

#### 4.2.2. Location Bias

The searches for relevant studies focusing on alexithymia and chronic tinnitus were also conducted within electronic databases. A total of three studies met inclusion criteria and were included. Two of these studies were conducted in the European Region and one in the South-East Asia Region. No included study was conducted within the African Region, Region of the Americas, Eastern Mediterranean Region, or Western Pacific Region, which leads to a possible location bias. As mentioned before, this location bias might be explained by the language restrictions—English or German publications—we imposed.

#### 4.2.3. Conclusions

Compiled results of the included studies show that THI scores were positively associated with the ‘emotion-focused’ TAS-20 total, DIF and DDF but not the ‘cognitiion-focused’ EOT scores. There appears to be no association between tinnitus-related distress and externally oriented thinking. This finding is in keeping with De Gucht et al. [37], who concluded that EOT was also unrelated to somatoform symptom presentations. The prevalence rate of alexithymia in a sample of chronic tinnitus patients, 65.7% [107], appears much higher than in samples of patients suffering from other psychological conditions like chronic pain, 47% [144], fibromyalgia, 15–52% [145], medically unexplained symptoms, 23.7% [146], and medically unexplained physical symptoms, 49.5% [147]. This implicates a possible relevance of alexithymia in chronic tinnitus presentations and suggests that therapeutic interventions might aim to assess and address individual alexithymic characteristics when treating chronic tinnitus-related distress.

The above-described findings reflect only three available cross-sectional studies. No longitudinal study focusing on alexithymia in cohorts suffering from chronic tinnitus could be found. Consequently, longitudinally designed studies are much needed to investigate the role of alexithymia in patients with chronic tinnitus.

### 4.3. Summary

Alexithymia plays an important role regarding interactions between emotional burden and bodily expressed symptoms. Addressing alexithymia psychotherapeutically improves psychological burden and bodily expressed symptoms. Initial studies indicate alexithymia as relevant intrapsychic factor for chronic tinnitus and tinnitus-related distress. Psychotherapeutic interventions lead to improvements in tinnitus-related distress and constitute its gold standard treatment [105]. Psychotherapy also improves stress [148], depressivity [123,126], and anxiousness [126]. Although some above-mentioned studies demonstrated that psychotherapeutic interventions led to improvements of alexithymia [71,120,122,123,126], it is still unclear whether alexithymia mediates outcomes across other symptom presentations. Once possible underlying medical conditions have been ruled out or treated as applicable, transdiagnostic psychological factors may play an important role for the psychotherapeutic conceptualization of tinnitus-related and broader emotional distress. In this context, alexithymia ought to be considered for psychotherapeutic formulation and treatment planning and addressed using emotion-focused interventions, as applicable. 

### 4.4. Limitations

The whole review process, including study selection, data extraction, and data synthesis, was conducted by one single researcher. This so-called single-screening or rapid review (RR) [149] may result in missing studies which might have been identified through conventional double-screening [150,151,152]. Implementation of a double-screening review process was not possible, so we decided to solve uncertainties by discussion in the author research team. Plüddemann et al. [153] view single-screening review processes as feasible and state they can be supplemented by including partial verification by a second-review member. Partial verification by an experienced second-review member was carried out when uncertainties within the whole review process arose (BB). Watt et al. [154] point out that when comparing both rapid and full systematic reviews directly, no extensive difference within conclusions can be found. Rapid reviews, conducted by a single researcher, thereby often provide appropriate guidance as a basis for clinical and policy decisions [154] in a timely and resource-efficient way [155]. Unsurprisingly, the number of conducted rapid reviews within the health care sector has increased in recent years [156].

Another important limitation concerns the as-yet small number of studies examining alexithymia in patients with chronic tinnitus. Future work needs to continue to examine the role of emotional avoidance or uncertainty in causing or maintaining tinnitus-related distress.

## Figures and Tables

**Table 1:** Diagnostic Criteria of Somatic Symptom Disorder within DSM-5 [43].

A	One or more somatic symptoms that are distressing or result in significant disruption of daily life.
B	Excessive thoughts, feelings, or behaviors related to the somatic symptoms or associated health concerns as manifested by at least one of the following: disproportionate and persistent thoughts about the seriousness of one’s symptoms. persistently high level of anxiety about health or symptoms. excessive time and energy devoted to these symptoms or health concerns.
C	Although any one somatic symptom may not be continuously present, the state of being symptomatic is persistent (typically more than 6 months).

Note. Severity is characterized by numbers of fulfilled symptoms under Criterion B. Mild: One symptom. Moderate: Two or more symptoms. Severe: Two or more symptoms, plus multiple somatic complaints (or one very severe somatic symptom).

**Table 2:** Comparison of Eligibility Criteria for Study Inclusion of both Parts of the current Review.

Review Part	Subjects	Assessment	Study Design	Publication
Part 1	adult human beings (≥18 years old) suffering from different kinds of somatization	assessment of alexithymia and somatization	longitudinal/prospective study designs	English or German; publication between 2001 and 2021
Part 2	adult human beings (≥18 years old) suffering from chronic tinnitus	assessment of alexithymia and chronic tinnitus	cross-sectional and longitudinal/prospective study designs	English or German; publication between 2001 and 2021

**Table 3:** Search Terms used within Databases focusing on Alexithymia and Somatoform Conditions.

Database	Defined Search Term
EBSCO	(((TI (alexithymia OR alexithym* OR (emotion* AND blindness) OR (emotion* AND deficiency))) AND (TI (somatization OR (somatization AND (disorder* OR condition*)) OR (somatoform AND (disorder* OR condition*)) OR (functional AND (disorder* OR condition*)) OR “functional somatic syndrome*” OR “undifferentiated somatoform disorder*” OR “undifferentiated somatoform condition*” OR “medically unexplained symptom*” OR “medically unexplained syndrome*” OR “psychovegetative syndrome*” OR “psychogenic disease*” OR “psychogenic illness*” OR “vegetative lability” OR “vegetative dystonia” OR “vegetative neuros*” OR “organ neuros*” OR hysteri* OR (hysteri* AND neuros*) OR (conversion AND (disorder* OR condition*)) OR (conversion AND neuros*) OR (chronic AND pain) OR “chronic pain disorder” OR “chronic pain condition”)))) AND ((AB (psychotherap* OR “CBT” OR “cognitive behavioral therapy” OR therap* OR psycho* OR counseling OR (cognitive AND therap*) OR (behavioral AND therap*) OR (cognitive AND psycho*) OR (behavioral AND psycho*) OR (psychodynamic AND therap*) OR (psychodynamic AND psycho*) OR (psychol* AND treatment*) OR (longitudinal AND stud*) OR (longitudinal AND research*) OR (longitudinal AND method*) OR (long* AND term*) OR longitudinal OR repeated-measure* OR (mixed AND model*) OR two-timepoint* OR multi-timepoint* OR *-timepoint* OR (baseline AND predictor*) OR (treatment AND effect*) OR (treatment AND efficacy) OR outcome* OR (treatment AND outcome*) OR (clinical AND effect*) OR (clinical AND efficacy) OR (clinical AND outcome*) OR effect*)) OR (SU (psychotherap* OR “CBT” OR “cognitive behavioral therapy” OR therap* OR psycho* OR counseling OR (cognitive AND therap*) OR (behavioral AND therap*) OR (cognitive AND psycho*) OR (behavioral AND psycho*) OR (psychodynamic AND therap*) OR (psychodynamic AND psycho*) OR (psychol* AND treatment*) OR (longitudinal AND stud*) OR (longitudinal AND research*) OR (longitudinal AND method*) OR (long* AND term*) OR longitudinal OR repeated-measure* OR (mixed AND model*) OR two-timepoint* OR multi-timepoint* OR *-timepoint* OR (baseline AND predictor*) OR (treatment AND effect*) OR (treatment AND efficacy) OR outcome* OR (treatment AND outcome*) OR (clinical AND effect*) OR (clinical AND efficacy) OR (clinical AND outcome*) OR effect*))))
EMBASE by Ovid	(((alexithymia.at. OR alexithym*.at. OR (emotion*.at. AND blindness.at.) OR (emotion*.at. AND deficiency.at.)) AND (somatization.at. OR (somatization.at. AND disorder*.at.) OR (somatoform.at. AND disorder*.at.) OR (functional.at. AND disorder*.at.) OR “functional somatic syndrome*”.at. OR “undifferentiated somatoform disorder*”.at. OR “medically unexplained symptom*”.at. OR “psychovegetative syndrome*”.at. OR “psychogenic disease*”.at. OR “psychogenic illness”.at. OR “vegetative lability”.at. OR “vegetative dystonia”.at. OR “vegetative neurosis”.at. OR “organ neurosis”.at. OR hysteri*.at. OR (hysteric*.at. AND neuros*.at.) OR (conversion.at. AND disorder*.at.) OR (conversion.at. AND neuros*.at.) OR (chronic.at. AND pain.at.) OR “chronic pain disorder”.at.)))) AND ((psychotherap*.ab. OR “CBT”.ab. OR “cognitive behavioral therapy”.ab. OR therap*.ab. OR psycho*.ab. OR counseling.ab. OR (cognitive.ab. AND therap*.ab.) OR (behavioral.ab. AND therap*.ab.) OR (cognitive.ab. AND psycho*.ab.) OR (behavioral.ab. AND psycho*.ab.) OR (psychodynamic.ab. AND therap*.ab.) OR (psychodynamic.ab. AND psycho*.ab.) OR (psychol*.ab. AND treatment.ab.) OR (longitudinal.ab. AND stud*.ab.) OR (longitudinal.ab. AND research*.ab.) OR (longitudinal.ab. AND method*.ab.) OR (long*.ab. AND term*.ab.) OR longitudinal.ab. OR repeated-measure*.ab. OR (mixed.ab. AND model*.ab.) OR two-timepoint*.ab. OR multi-timepoint*.ab. OR *-timepoint*.ab. OR (baseline.ab. AND predictor*.ab.) OR (treatment.ab. AND effect*.ab.) OR (treatment.ab. AND efficacy.ab.) OR outcome*.ab. OR (treatment.ab. AND outcome*.ab.) OR (clinical.ab. AND effect*.ab.) OR (clinical.ab. AND efficacy.ab.) OR (clinical.ab. AND outcome*.ab.) OR effect*.ab.) OR (psychotherap*.kw. OR “CBT”.kw. OR “cognitive behavioral therapy”.kw. OR therap*.kw. OR psycho*.kw. OR counseling.kw. OR (cognitive.kw. AND therap*.kw.) OR (behavioral.kw. AND therap*.kw.) OR (cognitive.kw. AND psycho*.kw.) OR (behavioral.kw. AND psycho*.kw.) OR (psychodynamic.kw. AND therap*.kw.) OR (psychodynamic.kw. AND psycho*.kw.) OR (psychol*.kw. AND treatment*.kw.) OR (longitudinal.kw. AND stud*.kw.) OR (longitudinal.kw. AND research.kw.) OR (longitudinal.kw. AND method*.kw.) OR (long*.kw. AND term*.kw.) OR longitudinal.kw. OR repeated-measure*.kw. OR (mixed.kw. AND model*.kw.) OR two-timepoint*.kw. OR multi-timepoint*.kw. OR *-timepoint*.kw. OR (baseline.kw. AND predictor*.kw.) OR (treatment.kw. AND effect*.kw.) OR (treatment.kw. AND efficacy.kw.) OR outcome*.kw. OR (treatment.kw. AND outcome*.kw.) OR (clinical.kw. AND effect*.kw.) OR (clinical.kw. AND efficacy.kw.) OR (clinical.kw. AND outcome*.kw.) OR effect*.kw.)))
PubMed	(((alexithymia[Title] OR alexithym*[Title] OR (emotion*[Title] AND blindness[Title]) OR (emotion*[Title] AND deficiency[Title])) AND (somatization[Title] OR (somatization[Title] AND disorder*[Title]) OR (somatoform[Title] AND disorder*[Title]) OR (functional[Title] AND disorder*[Title]) OR “functional somatic syndrome*”[Title] OR “undifferentiated somatoform disorder*”[Title] OR “medically unexplained symptom*”[Title] OR “medically unexplained syndrome*”[Title] OR “psychovegetative syndrome*”[Title] OR “psychogenic disease*”[Title] OR “psychogenic illness”[Title] OR “vegetative lability”[Title] OR “vegetative dystonia”[Title] OR “vegetative neurosis”[Title] OR “organ neurosis”[Title] OR hysteri*[Title] OR (hysteric*[Title] AND neuros*[Title]) OR (conversion[Title] AND disorder*[Title]) OR (conversion[Title] AND neuros*[Title]) OR (chronic[Title] AND pain[Title]) OR “chronic pain disorder”[Title])) AND ((psychotherap*[Title/Abstract] OR “CBT”[Title/Abstract] OR “cognitive behavioral therapy”[Title/Abstract] OR therap*[Title/Abstract] OR psycho*[Title/Abstract] OR counseling[Title/Abstract] OR (cognitive[Title/Abstract] AND therap*[Title/Abstract]) OR (behavioral[Title/Abstract] AND therap*[Title/Abstract]) OR (cognitive[Title/Abstract] AND psycho*[Title/Abstract]) OR (behavioral[Title/Abstract] AND psycho*[Title/Abstract]) OR (psychodynamic[Title/Abstract] AND therap*[Title/Abstract]) OR (psychodynamic[Title/Abstract] AND psycho*[Title/Abstract]) OR (psychol*[Title/Abstract] AND treatment[Title/Abstract]) OR (longitudinal[Title/Abstract] AND stud*[Title/Abstract]) OR (longitudinal[Title/Abstract] AND research*[Title/Abstract]) OR (longitudinal[Title/Abstract] AND method*[Title/Abstract]) OR (long*[Title/Abstract] AND term*[Title/Abstract]) OR longitudinal[Title/Abstract] OR repeated-measure*[Title/Abstract] OR (mixed[Title/Abstract] AND model*[Title/Abstract]) OR two-timepoint*[Title/Abstract] OR multi-timepoint*[Title/Abstract] OR *-timepoint*[Title/Abstract] OR (baseline[Title/Abstract] AND predictor*[Title/Abstract]) OR (treatment[Title/Abstract] AND effect*[Title/Abstract]) OR (treatment[Title/Abstract] AND efficacy[Title/Abstract]) OR outcome*[Title/Abstract] OR (treatment[Title/Abstract] AND outcome*[Title/Abstract]) OR (clinical[Title/Abstract] AND effect*[Title/Abstract]) OR (clinical[Title/Abstract] AND efficacy[Title/Abstract]) OR (clinical[Title/Abstract] AND outcome*[Title/Abstract]) OR effect*[Title/Abstract]) OR (psychotherap*[MeSH Terms] OR “CBT”[MeSH Terms] OR “cognitive behavioral therapy”[MeSH Terms] OR therap* OR psycho* OR counseling OR (cognitive[MeSH Terms] AND therap*[MeSH Terms]) OR (behavioral[MeSH Terms] AND therap*[MeSH Terms]) OR (cognitive[MeSH Terms] AND psycho*[MeSH Terms]) OR (behavioral[MeSH Terms] AND psycho*[MeSH Terms]) OR (psychodynamic[MeSH Terms] AND therap*[MeSH Terms]) OR (psychodynamic[MeSH Terms] AND psycho*[MeSH Terms]) OR (psychol*[MeSH Terms] AND treatment*[MeSH Terms]) OR (longitudinal[MeSH Terms] AND stud*[MeSH Terms]) OR (longitudinal[MeSH Terms] AND research*[MeSH Terms]) OR (longitudinal[MeSH Terms] AND method*[MeSH Terms]) OR (long*[MeSH Terms] AND term*[MeSH Terms]) OR longitudinal[MeSH Terms] OR repeated-measure*[MeSH Terms] OR (mixed[MeSH Terms] AND model*[MeSH Terms]) OR two-timepoint*[MeSH Terms] OR multi-timepoint*[MeSH Terms] OR *-timepoint*[MeSH Terms] OR (baseline[MeSH Terms] AND predictor*[MeSH Terms]) OR (treatment[MeSH Terms] AND effect*[MeSH Terms]) OR (treatment[MeSH Terms] AND efficacy[MeSH Terms]) OR outcome*[MeSH Terms] OR (treatment[MeSH Terms] AND outcome*[MeSH Terms]) OR (clinical[MeSH Terms] AND effect*[MeSH Terms]) OR (clinical[MeSH Terms] AND efficacy[MeSH Terms]) OR (clinical[MeSH Terms] AND outcome*[MeSH Terms]) OR effect*[MeSH Terms])))
Web of Science	(((TI = (alexithymia OR alexithym* OR (emotion* AND blindness) OR (emotion* AND deficiency))) AND (TI = (somatization OR (somatization AND disorder*) OR (somatoform AND disorder*) OR (functional AND disorder*) OR “functional somatic syndrome*” OR “undifferentiated somatoform disorder*” OR “medically unexplained symptom*” OR “medically unexplained syndrome*” OR “psychovegetative syndrome*” OR “psychogenic disease*” OR “psychogenic illness” OR “vegetative lability” OR “vegetative dystonia” OR “vegetative neurosis” OR “organ neurosis” OR hysteri* OR (hysteric* AND neuros*) OR (conversion AND disorder*) OR (conversion AND neuros*) OR (chronic pain) OR “chronic pain disorder”))) AND ((AB = (psychotherap* OR CBT OR “cognitive behavioral therapy” OR therapy OR psycho* OR counseling OR (cognitive AND therap*) OR (behavioral AND therap*) OR (cognitive AND psycho*) OR (behavioral AND psycho*) OR (psychodynamic AND therap*) OR (psychodynamic AND psycho*) OR (psychol* AND treatment) OR (longitudinal AND stud*) OR (longitudinal AND research*) OR (longitudinal AND method*) OR (long* AND term*) OR longitudinal OR repeated-measure* OR (mixed AND model*) OR two-timepoint* OR multi-timepoint* OR *-timepoint* OR (baseline AND predictor*) OR (treatment AND effect*) OR (treatment AND efficacy) OR outcome* OR (treatment AND outcome*) OR (clinical AND effect*) OR (clinical AND efficacy) OR (clinical AND outcome*) OR effect*)) OR (AK = (psychotherap* OR CBT OR “cognitive behavioral therapy” OR therapy OR psycho* OR counseling OR (cognitive AND therap*) OR (behavioral AND therap*) OR (cognitive AND psycho*) OR (behavioral AND psycho*) OR (psychodynamic AND therap*) OR (psychodynamic AND psycho*) OR (psychol* AND treatment) OR (longitudinal AND stud*) OR (longitudinal AND research*) OR (longitudinal AND method*) OR (long* AND term*) OR longitudinal OR repeated-measure* OR (mixed AND model*) OR two-timepoint* OR multi-timepoint* OR *-timepoint* OR (baseline AND predictor*) OR (treatment AND effect*) OR (treatment AND efficacy) OR outcome* OR (treatment AND outcome*) OR (clinical AND effect*) OR (clinical AND efficacy) OR (clinical AND outcome*) OR effect*))))

* Truncations were used for inclusivity of keywords with different endings.

**Table 4:** Search Terms used within Databases focusing on Alexithymia and Chronic Tinnitus.

Database	Defined Search Term
EBSCO	((TI (alexithymia OR alexithym* OR (emotion* AND blindness) OR (emotion* AND deficiency)) AND (TI (tinnit*s OR tinnitus OR tinnitus-related distress OR (ringing AND ear*) OR (buzzing AND ear*) OR (noise AND ear*) OR (hissing AND ear*) OR (humming AND ear*))))) OR ((AB (alexithymia OR (emotion* AND blindness) OR (emotion* AND deficiency)) AND (AB (tinnit*s OR tinnitus OR tinnitus-related distress OR (ringing AND ear*) OR (buzzing AND ear*) OR (noise AND ear*) OR (hissing AND ear*) OR (humming AND ear*))))) OR ((SU (alexithymia OR (emotion* AND blindness) OR (emotion* AND deficiency)) AND (AK = (tinnit*s OR tinnitus OR tinnitus-related distress OR (ringing AND ear*) OR (buzzing AND ear*) OR (noise AND ear*) OR (hissing AND ear*) OR (humming AND ear*)))))
EMBASE by Ovid	((alexithymia.at. OR alexithym* OR (emotion*.at. AND blindness.at.) OR (emotion*.at. AND deficiency.at.)) AND (tinnit*s.at. OR tinnitus.at. OR tinnitus-related distress.at. OR (ringing.at. AND ear*.at.) OR (buzzing.at. AND ear*.at.) OR (noise.at. AND ear*.at.) OR (hissing.at. AND ear*.at.) OR (humming.at. AND ear*.at.))) OR ((alexithymia.ab. OR (emotion*.ab. AND blindness.ab.) OR (emotion*.ab. AND deficiency.ab.)) AND (tinnit*s.ab. OR tinnitus.ab. OR tinnitus-related distress.ab. OR (ringing.ab. AND ear*.ab.) OR (buzzing.ab. AND ear*.ab.) OR (noise.ab. AND ear*.ab.) OR (hissing.ab. AND ear*.ab.) OR (humming.ab. AND ear*.ab.))) OR ((alexithymia.kw. OR (emotion*.kw. AND blindness.kw.) OR (emotion*.kw. AND deficiency.kw.)) AND (tinnit*s.kw. OR tinnitus.kw. OR tinnitus-related distress.kw. OR (ringing.kw. AND ear*.kw.) OR (buzzing.kw. AND ear*.kw.) OR (noise.kw. AND ear*.kw.) OR (hissing.kw. AND ear*.kw.) OR (humming.kw. AND ear*.kw.)))
PubMed	((alexithymia[Title/Abstract] OR alexithym*[Title/Abstract] OR (emotion*[Title/Abstract] AND blindness[Title/Abstract]) OR (emotion*[Title/Abstract] AND deficiency[Title/Abstract])) AND (tinnit*s[Title/Abstract] OR tinnitus[Title/Abstract] OR tinnitus-related distress[Title/Abstract] OR (ringing[Title/Abstract] AND ear*[Title/Abstract]) OR (buzzing[Title/Abstract] AND ear*[Title/Abstract]) OR (noise[Title/Abstract] AND ear*[Title/Abstract]) OR (hissing[Title/Abstract] AND ear*[Title/Abstract]) OR (humming[Title/Abstract] AND ear*[Title/Abstract]))) OR((alexithymia[MeSH Terms] OR (emotion*[MeSH Terms] AND blindness[MeSH Terms]) OR (emotion*[MeSH Terms] AND deficiency[MeSH Terms])) AND (tinnit*s[MeSH Terms] OR tinnitus[MeSH Terms] OR “tinnitus-related distress”[MeSH Terms] OR (ringing[MeSH Terms] AND ear*[MeSH Terms]) OR (buzzing[MeSH Terms] AND ear*[MeSH Terms]) OR (noise[MeSH Terms] AND ear*[MeSH Terms]) OR (hissing[MeSH Terms] AND ear*[MeSH Terms]) OR (humming[MeSH Terms] AND ear*[MeSH Terms]))
Web of Science	((TI = (alexithymia OR alexithym* OR (emotion* AND blindness) OR (emotion* AND deficiency)) AND (TI = (tinnit*s OR tinnitus OR tinnitus-related distress OR (ringing AND ear*) OR (buzzing AND ear*) OR (noise AND ear*) OR (hissing AND ear*) OR (humming AND ear*))))) OR ((AB = (alexithymia OR (emotion* AND blindness) OR (emotion* AND deficiency)) AND (AB = (tinnit*s OR tinnitus OR tinnitus-related distress OR (ringing AND ear*) OR (buzzing AND ear*) OR (noise AND ear*) OR (hissing AND ear*) OR (humming AND ear*))))) OR ((AK = (alexithymia OR (emotion* AND blindness) OR (emotion* AND deficiency)) AND (AK = (tinnit*s OR tinnitus OR tinnitus-related distress OR (ringing AND ear*) OR (buzzing AND ear*) OR (noise AND ear*) OR (hissing AND ear*) OR (humming AND ear*)))))

* Truncations were used for inclusivity of keywords with different endings.

**Table 5:** Characteristics of Included Studies.

Study Ref.	Authors and Year	Years of Data Collection	Country	Sample Size	Age	Gender
1	Aboussouan, Mandell, Johnson, Thompson and Huffman [123]	2011–2015	USA	116	18–70	F = 116
2	Melin, Thulesius and Persson [71]	2003–2005	Sweden	59	27–46	F = 52; M = 7
3	Saariaho, Saariaho, Mattila, Joukamaa and Karukivi [124]	2004–2005	Finland	83	18–65	F = 49; M = 34
4	Saariaho, Saariaho, Mattila, Ohtonen, Joukamaa and Karukivi [125]	2004–2005	Finland	154	18–65	F = 86; M = 68
5	Porcelli, Bagby, Taylor, De Carne, Leandro and Todarello [126]	1997–1998	Italy	130	NR	F = 86; M = 44
6	Reese [122]	NR	USA	84	22–65	F = 75; M = 9
7	Probst, Sattel, Gündel, Henningsen, Kruse, Schneider and Lahmann [121]	NR	Germany	83	NR	F = 50; M = 33
8	Saedi, Hatami, Asgari, Ahadi and Poursharifi [120]	NR	Iran	30	20–50	NR

NR: not reported; F: female; M: male.

**Table 6:** Analyses conducted within Included Studies.

Study Ref.	Authors and Year	Associations between Alexithymia and Other Baseline Variables	Alexithymia as Outcome Variable	Associations between Alexithymia and Non-Alexithymia-Related Treatment Outcomes
1	Aboussouan, Mandell, Johnson, Thompson and Huffman [123] *		✓	✓
2	Melin, Thulesius and Persson [71] *	✓	✓	✓
3	Saariaho, Saariaho, Mattila, Joukamaa and Karukivi [124] *	✓	✓	✓
4	Saariaho, Saariaho, Mattila, Ohtonen, Joukamaa and Karukivi [125] *		✓	✓
5	Porcelli, Bagby, Taylor, De Carne, Leandro and Todarello [126] *		✓	✓
6	Reese [122]		✓	✓
7	Probst, Sattel, Gündel, Henningsen, Kruse, Schneider and Lahmann [121] *			✓
8	Saedi, Hatami, Asgari, Ahadi and Poursharifi [120]		✓	✓

* Studies that controlled for depression within analyses.

**Table 7:** Results of Risk of Bias Assessment for RCTs by Rob 2 [129].

RCTs	D1	D2	D3	D4	D5	Overall
Reese [122]						
Probst, Sattel, Gündel, Henningsen, Kruse, Schneider and Lahmann [121]						
Saedi, Hatami, Asgari, Ahadi and Poursharifi [120]						

D1: randomization process, D2: deviations from intended interventions, D3: missing outcome data, D4: measurement of the outcome, D5: selection of the reported result; green: low risk, yellow: some concerns, red: high risk.

**Table 8:** Results of Risk of Bias Assessment for NRCTs by ROBINS-I [127].

RCTs	D1	D2	D3	D4	D5	D6	D7	Overall
Aboussouan, Mandell, Johnson, Thompson and Huffman [123]								
Melin, Thulesius and Persson [71]					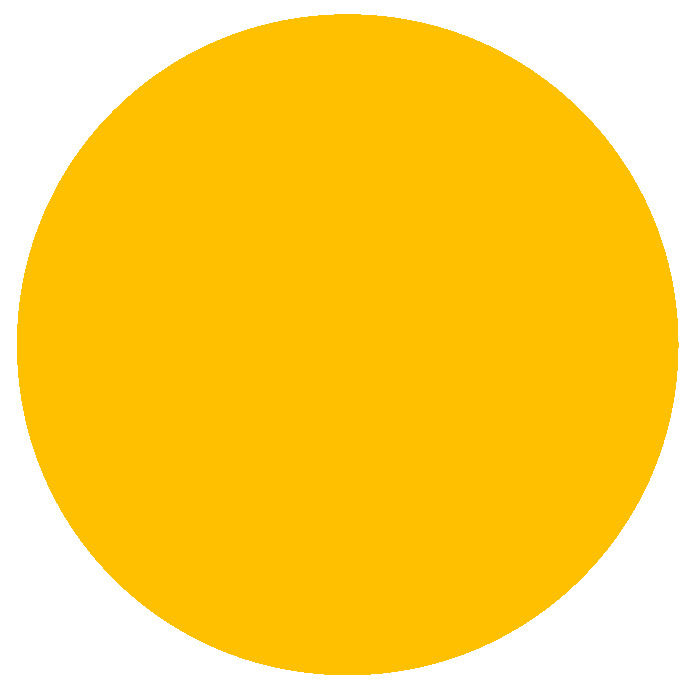			
Saariaho, Saariaho, Mattila, Joukamaa and Karukivi [124]			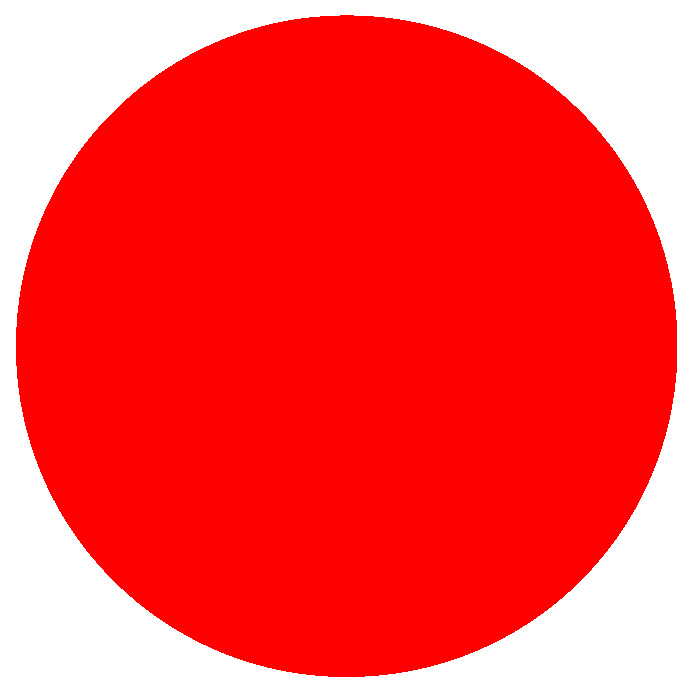					
Saariaho, Saariaho, Mattila, Ohtonen, Joukamaa and Karukivi [125]			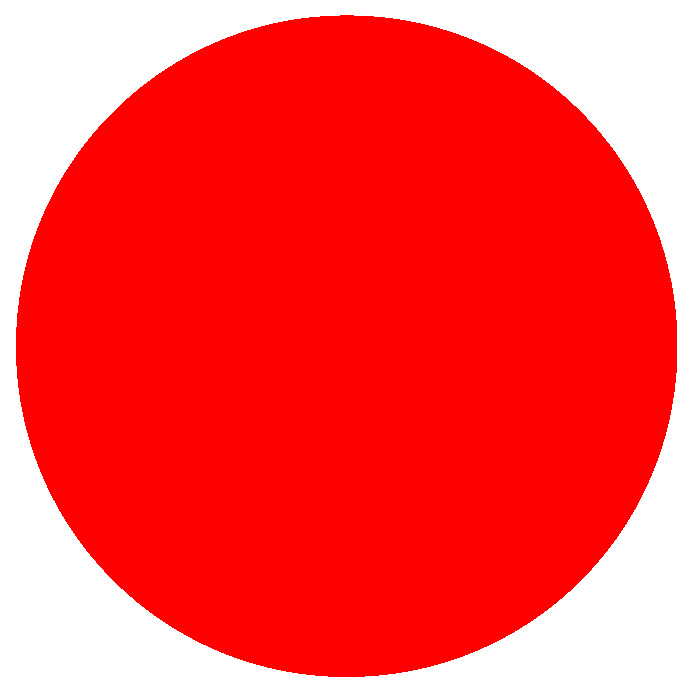					
Porcelli, Bagby, Taylor, De Carne, Leandro and Todarello [126]								

D1: confounding, D2: selection of participants into the study, D3: classification of interventions, D4: deviations from intended interventions, D5: missing data, D6: measurement of outcomes, D7: selection of the reported result; green: low risk, yellow: moderate risk, orange: serious risk, red: critical risk.

**Table 9:** Characteristics of Included Studies.

Study Ref.	Authors and Year	Years of Data Collection	Country	Study Design	Sample Size	Age	Gender
1	Bakhla, Dayal, Bala and Toppo [107]	NR	India	cross-sectional	70	Mean = 33.17 ± 12.24	F = 31 M = 39
2	Salonen, Johansson and Joukamaa [109]	2002–2003	Finland	cross-sectional	583	71–86	F = 343 M = 240
3	Wielopolski, Kleinjung, Koch, Peter, Meyer, Rufer and Weidt [108]	2012–2014	Switzerland	cross-sectional	207	Mean = 46.7 (SD = 13.9)	F = 73 M = 134

NR: not reported; F: female; M: male.

**Table 10:** Analyses conducted within Included Studies.

Study Ref.	Authors and Year	Prevalence Rates of Alexithymia in Patients with Chronic Tinnitus	Associations between Alexithymia and Tinnitus-Related Distress	Differences in Alexithymia in Patients with Bothersome versus Non-Bothersome Tinnitus
1	Bakhla, Dayal, Bala and Toppo [107]	✓	✓	
2	Salonen, Johansson and Joukamaa [109]			✓
3	Wielopolski, Kleinjung, Koch, Peter, Meyer, Rufer and Weidt [108]	✓	✓	

**Table 11:** Assessment of Risk of Bias with JBI Critical Appraisal Checklist for Analytical Cross Sectional Studies [130].

Reference	Signaling Question	Response
Bakhla, Dayal, Bala and Toppo [107]	1. Were the criteria for inclusion in the sample clearly defined?	Y
	2. Were the study subjects and the setting described in detail?	Y
	3. Was the exposure measured in a valid and reliable way?	Y
	4. Were objective, standard criteria used for measurement of the condition?	Y
	5. Were confounding factors identified?	Y
	6. Were strategies to deal with confounding factors stated?	N
	7. Were the outcomes measured in a valid and reliable way?	Y
	8. Was appropriate statistical analysis used?	Y
Salonen, Johansson and Joukamaa [109]	1. Were the criteria for inclusion in the sample clearly defined?	Y
	2. Were the study subjects and the setting described in detail?	Y
	3. Was the exposure measured in a valid and reliable way?	Y
	4. Were objective, standard criteria used for measurement of the condition?	Y
	5. Were confounding factors identified?	Y
	6. Were strategies to deal with confounding factors stated?	N
	7. Were the outcomes measured in a valid and reliable way?	Y
	8. Was appropriate statistical analysis used?	Y
Wielopolski, Kleinjung, Koch, Peter, Meyer, Rufer and Weidt [108]	1. Were the criteria for inclusion in the sample clearly defined?	Y
	2. Were the study subjects and the setting described in detail?	Y
	3. Was the exposure measured in a valid and reliable way?	Y
	4. Were objective, standard criteria used for measurement of the condition?	Y
	5. Were confounding factors identified?	Y
	6. Were strategies to deal with confounding factors stated?	N
	7. Were the outcomes measured in a valid and reliable way?	Y
	8. Was appropriate statistical analysis used?	Y

Y: yes, N: no.

## Data Availability

All relevant data are within this manuscript and its supplementary files.

## Supplementary Materials

The following supporting information can be downloaded at: https://www.mdpi.com/article/10.3390/jcm12216828/s1, Table S1: Overview of Included Treatment Studies focusing on Alexithymia and Somatoform Conditions. Table S2: Overview of Included Studies focusing on Alexithymia and Chronic Tinnitus. Table S3: Assessment of risk of bias for RCTs focusing on Alexithymia and Somatization. Table S4: Assessment of risk of bias for Non-RCTs focusing on Alexithymia and Somatization.
