# Supplementary material for: Alexithymia in Patients with Somatization Difficulties and Tinnitus-Related Distress: A Systematic Review
Source: J Clin Med. 2023 Oct 29;12(21):6828. doi: 10.3390/jcm12216828 (PMC10649228; doi:10.3390/jcm12216828)
Supplement: Supplementary file 1 [file jcm-12-06828-s001.zip › SupplementaryTable S1.pdf]

Supplementary Table S1. Overview of Included Treatment Studies focusing on Alexithymia and Somatoform Conditions

ID	Author(s) & Year	Title	Measurement timepoints	Type of treatment	Outcome measurement	Study outcome
1	Aboussouan, Mandell, Johnson, Thompson and Huffman [128]	An interdisciplinary chronic pain rehabilitation program effectively treats impairment in sexual function, depression, alexithymia, and pain in women with chronic pelvic pain	- baseline<br>- post-treatment	3-4 week intensive interdisciplinary program consisting of medication management, psychotherapy (individual, family, group), psychoeducation, physical and occupational therapy, weaning from habituating medications and optional monthly aftercare	- TAS-20<br>- PDI<br>- DASS-21<br>- NRS-11	- improvements in all outcome variables<br>- sexual function (Δ = 3.75, *SE* = 0.27)<br>- depression (Δ =13.86, *SE* = 1.16)<br>- alexithymia (Δ = 6.17, *SE* = 1.19)<br>- pain (Δ = 3.45, *SE* = 0.23)<br>- changes in alexithymia, depressivity and pain severity were significant predictors for post treatment impairment in sexual function and chronic pelvic pain
2	Melin, Thulesius and Persson [76]	Affect School for chronic benign pain patients showed improved alexithymia assessments with TAS-20	- baseline<br>- post-treatment	Affect School consisting of 8 sessions of group psychotherapy and 10 sessions of individual Script Analyses	- TAS-20<br>- HADS<br>- VAS-pain<br>- EQoL<br>- SCI-93	- higher baseline TAS-20 scores predicted significantly higher baseline depression, anxiety and stress symptoms<br>- TAS-20, DIF and DDF improved significantly, EOT did not<br>- significant post treatment improvements of alexithymia, depression and quality of life
3	Saariaho, Saariaho, Mattila, Joukamaa and Karukivi [129]	The role of alexithymia: An eight-year follow-up study of chronic pain patients	- baseline<br>- 8-year-follow-up	treatment protocols based on biomedical concepts and recommendations	- TAS-20<br>- BDI-II<br>- PDS<br>- VAS	- pain intensity, pain disability and depressivity decreased significantly<br>- no significant longitudinal change in alexithymia<br>- alexithymic subjects reported significantly more pain disability and depressivity at baseline, and more pain intensity, pain disability and depressivity at follow-up<br>- non-improvements in pain intensity were significantly associated with male gender, baseline TAS-20 and DDF
4	Saariaho, Saariaho, Mattila, Ohtonen, Joukamaa and Karukivi [130]	Alexithymia and depression in the recovery of chronic pain patients: a follow-up study	- baseline<br>- 1-year-follow-up	pain treatment methods consisting of invasive methods (anaesthesiological procedures / acupuncture) and non-invasive methods (drug therapy, physiotherapy, psychotherapy, pain groups)	- TAS-20<br>- BDI-II<br>- PDS<br>- VAS	- significant decrease of pain intensity, pain disability at follow-up<br>- TAS-20, DDF, and DIF increased significantly at follow-up in whole sample<br>- alexithymic patients reported significantly more pain disability and depression at both baseline and follow-up<br>- baseline TAS-20 was identified as significant predictor for pain disability<br>- BDI mediated between baseline TAS-20 and pain disability at follow-up

5	Porcelli, Bagby, Taylor, De Carne, Leandro and Todarello [131]	Alexithymia as Predictor of Treatment Outcome in Patients with Functional Gastrointestinal Disorders	- baseline<br>- 6-months-follow-up	personalized treatment consisting of drugs, diet modifications, psychotropic medications, psychological counseling or brief psychotherapy	- TAS-20<br>- HADS<br>- GSRS	- unimproved patients reported significant higher scores of TAS-20, HADS-A, HADS-D and GSRS at baseline<br>- TAS-20 correlated significantly with GSRS at baseline and follow-up<br>- changes in TAS-20, HADS-A, HADS-D, GSRS were significant within patients improved in functional gastrointestinal disorder; unimproved just TAS-20 changed significantly<br>- baseline TAS-20 significantly predicted TAS-20 at follow-up even when controlling for HADS-A and HADS-D baseline and follow-up scores<br>- TAS-20 strongest assessed single predictor for treatment outcome<br>- TAS-20 baseline scores increased prediction of variance of HADS-A, HADS-D and GSRS significantly
6	Reese [127]	Predicting improvement in Cognitive Behavioral Therapy for somatization disorder: The role of alexithymia	- baseline<br>- 3-months-follow-up (post baseline)<br>- 9-months-follow-up (post baseline)<br>- 15-months-follow-up (post baseline)	10-session individual CBT in combination of a psychiatric consultation letter (PCL)	- TAS-20<br>- CGI-SD<br>- Daily Symptom Diary<br>- SF-36<br>- Marlowe-Crowne Social Desirability Scale<br>- SSAS	- TAS-20 highly stable over time<br>- alexithymic subjects reported poorer mental health, more anxiousness and lower defensiveness at baseline, and more anxiousness and better physical functioning at follow-up<br>- defensiveness negatively correlated with DIF and DDF<br>- changes in TAS-20, DIF, DDF significantly correlated to improvement of somatization symptoms (CGI-SD) and physical functioning at post-treatment and 12months-follow-up<br>- EOT changes significantly correlated to CGI-SD improvement at post-treatment<br>- EOT significant correlated to duration of symptoms<br><br><u>changes of alexithymia</u><br>- TAS-20 score significant time-condition interaction<br>- CBT+PCL significantly changed more in DIF than PCL<br>- treatment groups changed significantly in TAS-20, DIF and EOT<br><br><u>Intent-to-treat</u><br>- high defensiveness + high anxiety significantly scored higher on TAS-20 at baseline

- high anxiety scored significantly higher on DIF at baseline
- repressors = greater mental health than groups with high anxiety
- low anxiety + low defensiveness greater mental health at baseline than low defensiveness + high anxiety
- high defensiveness + high anxiety and low defensiveness + high anxiety scored higher in TAS-20 than repressors
- Follow-up: high defensiveness + high anxiety scored higher on alexithymia than repressors
- Post-treatment: defensiveness + high anxiety and low defensiveness + high anxiety scored higher on DIF than repressors
- Follow-up: high defensiveness + high anxiety scored higher on DIF
- Post-treatment: Low defensiveness + high anxiety scored higher on DDF than repressors

<u>Mediational Analyses</u>
- treatment condition significantly predicted improvement in somatization symptoms and improvement in changes of TAS-20, DIF and EOT at post-treatment; improvement in somatization symptoms was significantly predicted by changes in TAS-20, DIF and EOT
- treatment condition significantly predicted greater physical functioning at post-treatment; physical functioning was also predicted by changes in TAS-20, DIF

- treatment condition significantly predicted improvement in somatization symptoms at 12-months-follow-up; improvement in somatization symptoms was significantly predicted by changes in TAS-20, DIF and DDF
- treatment condition significantly predicted greater physical functioning at 12-months-follow-up; changes in TAS-20 and DIF additionally predicted physical functioning

- treatment predicted daily diary scores; changes in EOT predicted diary scores at post-treatment; treatment predicted diary scores at 12-months-follow-up

7	Probst, Sattel, Gündel, Henningsen, Kruse, Schneider and Lahmann [126]	Moderating Effects of Alexithymia on Associations between the Therapeutic Alliance and the Outcome of Brief Psychodynamic-Interpersonal Psychotherapy for Multisomatoform Disorder	- baseline<br>- 9-months-follow-up (after treatment)	manualized brief psychodynamic-interpersonal therapy (PIT) consisting of 12 individual sessions (establishment of therapeutic alliance, treatment of somatoform symptoms, behavioral, emotional, and interpersonal correlates, termination issues)	- TAS-20<br>- SF-36<br>- HAQ<br>- PHQ-9	- TAS-20 scores were significantly positively associated with PHQ-9<br>- interaction effect between patients' alliance ratings and patients' alexithymia on physical quality of life at 9-month after treatment did not attain statistical significance, even after controlling for depression<br>- interaction effect between the therapists' alliance ratings and patients' alexithymia on physical quality of life at 9-month after PIT reached statistical significance, after controlling for depression this interaction effect did not reach significance
8	Saedi, Hatami, Asgari, Ahadi and Poursharifi [125]	The Effectiveness of Cognitive-Behavioral Therapy on Alexithymia and Pain. Self-Efficacy of Patients with Chronic Pain	- Baseline<br>- Post-treatment<br>- 3-months-follow-up	behavioral-cognitive training consisting of 8 sessions of 90 minutes	- Case-finding chronic pain questionnaire<br>- TAS-20<br>- PSEQ	- significant difference between patients within CBT and control group in terms of alexithymia and pain self-efficacy [$p \leq 0.001$]<br>- CBT led to increased self-efficacy of pain, reduced alexithymia, and harmful effects of pain
