# Supplementary material for: Alexithymia in Patients with Somatization Difficulties and Tinnitus-Related Distress: A Systematic Review
Source: J Clin Med. 2023 Oct 29;12(21):6828. doi: 10.3390/jcm12216828 (PMC10649228; doi:10.3390/jcm12216828)
Supplement: Supplementary file 1 [file jcm-12-06828-s001.zip › SupplementaryTable S2.pdf]

Supplementary Table S2. Overview of Included Studies focusing on Alexithymia and Chronic Tinnitus

ID	Author(s) & Year	Title	Outcome measurement	Study outcome
1	Bakhla, Dayal, Bala and Toppo [112]	Auditory sensation with affective agnosia: A prevalence of alexithymia among tinnitus patients	- TAS-20<br>- THI<br>- HADS-A<br>- HADS-D	- prevalence of tinnitus-related distress: slight (10%), mild (17.1%), moderate (20%), severe (34.3%) and catastrophic (18.6%)<br>- prevalence of alexithymia in patients suffering from tinnitus = 65.7%<br>- subgroup categorized as high THI significantly scored higher on TAS-20, DIF, DDF but not EOT and higher on depression and anxiety
2	Salonen, Johansson and Joukamaa [114]	Alexithymia, depression and tinnitus in elderly people	- TAS-20<br>- BDI<br>- *Individual* Tinnitus Questionnaire<br>- Audiometric measurements	- severity of alexithymia was associated with presence of tinnitus<br>- TAS-20 did not correlate with tinnitus severity (categorized as patients with or without annoyance due to tinnitus)<br>- patients with tinnitus but without annoyance were most commonly alexithymic and scored the highest on TAS-20<br>- patients with annoying tinnitus were more commonly alexithymic than subjects without tinnitus
3	Wielopolski, Kleinjung, Koch, Peter, Meyer, Rufer and Weidt [113]	Alexithymia Is Associated with Tinnitus Severity	- TAS-20<br>- THI<br>- BDI	- prevalence of tinnitus-related distress: slight (12.6%), mild (29%), moderate (28%), severe (21.7%) and catastrophic (8.7%)<br>- THI and its subscales (functional, emotional, catastrophic) were significantly associated with TAS-20, DIF and DDF but not EOT<br>- significant prediction of tinnitus-related distress by DIF and BDI<br>- THI functional subscale independently associated with higher DIF
