# Supplementary material for: Alexithymia in Patients with Somatization Difficulties and Tinnitus-Related Distress: A Systematic Review
Source: J Clin Med. 2023 Oct 29;12(21):6828. doi: 10.3390/jcm12216828 (PMC10649228; doi:10.3390/jcm12216828)
Supplement: Supplementary file 1 [file jcm-12-06828-s001.zip › SupplementaryTable S3.pdf]

Supplementary Table S3. Assessment of risk of bias for RCTs focusing on Alexithymia and Somatization

Reference	Domain	Signaling question	Response
Probst et al. (2017)	Bias arising from the randomization process	1.1 Was the allocation sequence random?	Y
		1.2 Was the allocation sequence concealed until participants were enrolled and assigned to interventions?	NI
		1.3 Did baseline differences between intervention groups suggest a problem with the randomization process?	NI
		Risk of bias judgement	Some concerns
	Bias due to deviations from intended interventions	2.1. Were participants aware of their assigned intervention during the trial?	NI
		2.2. Were carers and people delivering the interventions aware of participants' assigned intervention during the trial?	Y
		2.3. If Y/PY/NI to 2.1 or 2.2: Were there deviations from the intended intervention that arose because of the experimental context?	N
		2.4 If Y/PY to 2.3: Were these deviations likely to have affected the outcome?	NA
		2.5. If Y/PY/NI to 2.4: Were these deviations from intended intervention balanced between groups?	NA
		2.6 Was an appropriate analysis used to estimate the effect of assignment to intervention?	PY
		2.7 If N/PN/NI to 2.6: Was there potential for a substantial impact (on the result) of the failure to analyse participants in the group to which they were randomized?	NA
		Risk of bias judgement	Low
	Bias due to missing outcome data	3.1 Were data for this outcome available for all, or nearly all, participants randomized?	PY
		3.2 If N/PN/NI to 3.1: Is there evidence that result was not biased by missing outcome data?	NA
		3.3 If N/PN to 3.2: Could missingness in the outcome depend on its true value?	NA
		3.4 If Y/PY/NI to 3.3: Is it likely that missingness in the outcome depended on its true value?	NA
		Risk of bias judgement	Low
	Bias in measurement of the outcome	4.1 Was the method of measuring the outcome inappropriate?	N
		4.2 Could measurement or ascertainment of the outcome have differed between intervention groups?	PY
		4.3 Were outcome assessors aware of the intervention received by study participants?	NA
		4.4 If Y/PY/NI to 4.3: Could assessment of the outcome have been influenced by knowledge of intervention received?	NA
		4.5 If Y/PY/NI to 4.4: Is it likely that assessment of the outcome was influenced by knowledge of intervention received?	NA
		Risk of bias judgement	Some concerns
	Bias in selection of the reported result	5.1 Were the data that produced this result analysed in accordance with a pre-specified analysis plan that was finalized before unblinded outcome data were available for analysis?	PY
		5.2 ... multiple eligible outcome measurements (e.g. scales, definitions, time points) within the outcome domain?	PN
		5.3 ... multiple eligible analyses of the data?	N

		Risk of bias judgement	Low
	Overall bias	Risk of bias judgement	Low
Saedi et al. (2016)	Bias arising from the randomization process	1.1 Was the allocation sequence random?	Y
		1.2 Was the allocation sequence concealed until participants were enrolled and assigned to interventions?	NI
		1.3 Did baseline differences between intervention groups suggest a problem with the randomization process?	N
		Risk of bias judgement	Some concerns
	Bias due to deviations from intended interventions	2.1. Were participants aware of their assigned intervention during the trial?	PY
		2.2. Were carers and people delivering the interventions aware of participants' assigned intervention during the trial?	PY
		2.3. If Y/PY/NI to 2.1 or 2.2: Were there deviations from the intended intervention that arose because of the experimental context?	PN
		2.4 If Y/PY to 2.3: Were these deviations likely to have affected the outcome?	NA
		2.5. If Y/PY/NI to 2.4: Were these deviations from intended intervention balanced between groups?	NA
		2.6 Was an appropriate analysis used to estimate the effect of assignment to intervention?	PY
		2.7 If N/PN/NI to 2.6: Was there potential for a substantial impact (on the result) of the failure to analyse participants in the group to which they were randomized?	NA
		Risk of bias judgement	Low
	Bias due to missing outcome data	3.1 Were data for this outcome available for all, or nearly all, participants randomized?	Y
		3.2 If N/PN/NI to 3.1: Is there evidence that result was not biased by missing outcome data?	NA
		3.3 If N/PN to 3.2: Could missingness in the outcome depend on its true value?	NA
		3.4 If Y/PY/NI to 3.3: Is it likely that missingness in the outcome depended on its true value?	NA
		Risk of bias judgement	Low
	Bias in measurement of the outcome	4.1 Was the method of measuring the outcome inappropriate?	PN
		4.2 Could measurement or ascertainment of the outcome have differed between intervention groups?	PN
		4.3 Were outcome assessors aware of the intervention received by study participants?	PY
		4.4 If Y/PY/NI to 4.3: Could assessment of the outcome have been influenced by knowledge of intervention received?	PN
		4.5 If Y/PY/NI to 4.4: Is it likely that assessment of the outcome was influenced by knowledge of intervention received?	NA
		Risk of bias judgement	Low
	Bias in selection of the reported result	5.1 Were the data that produced this result analysed in accordance with a pre-specified analysis plan that was finalized before unblinded outcome data were available for analysis?	PY
		5.2 ... multiple eligible outcome measurements (e.g. scales, definitions, time points) within the outcome domain?	PN
		5.3 ... multiple eligible analyses of the data?	PN
		Risk of bias judgement	Low

	Overall bias	Risk of bias judgement	Some concerns
Reese (2008)	Bias arising from the randomization process	1.1 Was the allocation sequence random?	Y
		1.2 Was the allocation sequence concealed until participants were enrolled and assigned to interventions?	NI
		1.3 Did baseline differences between intervention groups suggest a problem with the randomization process?	PN
		Risk of bias judgement	Some concerns
	Bias due to deviations from intended interventions	2.1. Were participants aware of their assigned intervention during the trial?	PY
		2.2. Were carers and people delivering the interventions aware of participants' assigned intervention during the trial?	PY
		2.3. If Y/PY/NI to 2.1 or 2.2: Were there deviations from the intended intervention that arose because of the experimental context?	PN
		2.4 If Y/PY to 2.3: Were these deviations likely to have affected the outcome?	NA
		2.5. If Y/PY/NI to 2.4: Were these deviations from intended intervention balanced between groups?	NA
		2.6 Was an appropriate analysis used to estimate the effect of assignment to intervention?	Y
		2.7 If N/PN/NI to 2.6: Was there potential for a substantial impact (on the result) of the failure to analyse participants in the group to which they were randomized?	NA
		Risk of bias judgement	Low
	Bias due to missing outcome data	3.1 Were data for this outcome available for all, or nearly all, participants randomized?	PY
		3.2 If N/PN/NI to 3.1: Is there evidence that result was not biased by missing outcome data?	NA
		3.3 If N/PN to 3.2: Could missingness in the outcome depend on its true value?	NA
		3.4 If Y/PY/NI to 3.3: Is it likely that missingness in the outcome depended on its true value?	NA
		Risk of bias judgement	Low
	Bias in measurement of the outcome	4.1 Was the method of measuring the outcome inappropriate?	N
		4.2 Could measurement or ascertainment of the outcome have differed between intervention groups?	PN
		4.3 Were outcome assessors aware of the intervention received by study participants?	PY
		4.4 If Y/PY/NI to 4.3: Could assessment of the outcome have been influenced by knowledge of intervention received?	NI
		4.5 If Y/PY/NI to 4.4: Is it likely that assessment of the outcome was influenced by knowledge of intervention received?	PN
		Risk of bias judgement	Some concerns
	Bias in selection of the reported result	5.1 Were the data that produced this result analysed in accordance with a pre-specified analysis plan that was finalized before unblinded outcome data were available for analysis?	PY
		5.2 ... multiple eligible outcome measurements (e.g. scales, definitions, time points) within the outcome domain?	PN
		5.3 ... multiple eligible analyses of the data?	PN

	Risk of bias judgement	Low
Overall bias	Risk of bias judgement	Low

Y: yes; PY: probably yes; PN: probably no; N: no; NI: no information; NA: no answwe
