# Supplementary material for: Alexithymia in Patients with Somatization Difficulties and Tinnitus-Related Distress: A Systematic Review
Source: J Clin Med. 2023 Oct 29;12(21):6828. doi: 10.3390/jcm12216828 (PMC10649228; doi:10.3390/jcm12216828)
Supplement: Supplementary file 1 [file jcm-12-06828-s001.zip › SupplementaryTable S4.pdf]

Supplementary Table S4. Assessment of risk of bias for Non-RCTs focusing on Alexithymia and Somatization

Reference	Domain	Signaling question	Response
Aboussouan, Mandell, Johnson, Thompson and Huffman [128]	Bias due to confounding	1.1 Is there potential for confounding of the effect of intervention in this study?	Y
		If N/PN to 1.1: the study can be considered to be at low risk of bias due to confounding and no further signalling questions need be considered	
		If Y/PY to 1.1: determine whether there is a need to assess time-varying confounding:	
		1.2 Was the analysis based on splitting participants' follow up time according to intervention received?	N
		If N/PN, answer questions relating to baseline confounding (1.4 to 1.6)	
		If Y/PY, go to question 1.3.	
		1.3 Were intervention discontinuations or switches likely to be related to factors that are prognostic for the outcome?	NA
		If N/PN, answer questions relating to baseline confounding (1.4 to 1.6)	
		If Y/PY, answer questions relating to both baseline and time-varying confounding (1.7 and 1.8)	
		1.4. Did the authors use an appropriate analysis method that controlled for all the important confounding domains?	Y
		1.5. If Y/PY to 1.4: Were confounding domains that were controlled for measured validly and reliably by the variables available in this study?	PY
		1.6. Did the authors control for any post-intervention variables that could have been affected by the intervention?	N
		1.7. Did the authors use an appropriate analysis method that controlled for all the important confounding domains and for time-varying confounding?	Y
		1.8. If Y/PY to 1.7: Were confounding domains that were controlled for measured validly and reliably by the variables available in this study?	Y
		Risk of bias judgement	Moderate
	Bias in selection of participants into the study	2.1. Was selection of participants into the study (or into the analysis) based on participant characteristics observed after the start of intervention?	N
		2.2. If Y/PY to 2.1: Were the post-intervention variables that influenced selection likely to be associated with intervention?	NA
		2.3. If Y/PY to 2.2: Were the post-intervention variables that influenced selection likely to be influenced by the outcome or a cause of the outcome?	NA
		2.4. Do start of follow-up and start of intervention coincide for most participants?	Y
		2.5. If Y/PY to 2.2 and 2.3, or N/PN to 2.4: Were adjustment techniques used that are likely to correct for the presence of selection biases?	NA
		Risk of bias judgement	Low
	Bias in classification	3.1. Were intervention groups clearly defined?	Y
		3.2. Was the information used to define intervention groups recorded at the start of the intervention?	Y
		3.3. Could classification of intervention status have been affected by knowledge of the outcome or risk of the outcome?	PN

	of interventions	Risk of bias judgement	Low
	Bias due to deviations from intended interventions	4.1. Were there deviations from the intended intervention beyond what would be expected in usual practice?	N
		4.2. If Y/PY to 4.1: Were these deviations from intended intervention unbalanced between groups and likely to have affected the outcome?	NA
		If your aim for this study is to assess the effect of starting and adhering to intervention, answer questions 4.3 to 4.6	
		4.3. Were important co-interventions balanced across intervention groups?	NA
		4.4. Was the intervention implemented successfully for most participants?	NA
		4.5. Did study participants adhere to the assigned intervention regimen?	NA
		4.6. If N/PN to 4.3, 4.4 or 4.5: Was an appropriate analysis used to estimate the effect of starting and adhering to the intervention?	NA
		Risk of bias judgement	Low
	Bias due to missing data	5.1. Were outcome data available for all, or nearly all, participants?	Y
		5.2. Were participants excluded due to missing data on intervention status?	Y
		5.3. Were participants excluded due to missing data on other variables needed for the analysis?	Y
		5.4. If PN/N to 5.1, or Y/PY to 5.2 or 5.3: Are the proportion of participants and reasons for missing data similar across interventions?	PY
		5.5. If PN/N to 5.1, or Y/PY to 5.2 or 5.3: Is there evidence that results were robust to the presence of missing data?	NI
		Risk of bias judgement	Moderate
	Bias in measurement of outcomes	6.1. Could the outcome measure have been influenced by knowledge of the intervention received?	PN
		6.2. Were outcome assessors aware of the intervention received by study participants?	Y
		6.3. Were the methods of outcome assessment comparable across intervention groups?	Y
		6.4. Were any systematic errors in measurement of the outcome related to intervention received?	PN
		Risk of bias judgement	Moderate
	Bias in selection of the reported result	Is the reported effect estimate likely to be selected, on the basis of the results, from...	
		7.1. ... multiple outcome measurements within the outcome domain?	PN
		7.2. ... multiple analyses of the intervention-outcome relationship?	PN
		7.3. ... different subgroups?	PN
		Risk of bias judgement	Low
	**Overall bias**	Risk of bias judgement	Moderate
Melin, Thulesius	Bias due to confounding	1.1 Is there potential for confounding of the effect of intervention in this study?	Y
		If N/PN to 1.1: the study can be considered to be at low risk of bias due to confounding and no further signalling questions need be considered	

and Persson [76]		If Y/PY to 1.1: determine whether there is a need to assess time-varying confounding:	
		1.2 Was the analysis based on splitting participants' follow up time according to intervention received?	N
		If N/PN, answer questions relating to baseline confounding (1.4 to 1.6)	
		If Y/PY, go to question 1.3.	
		1.3 Were intervention discontinuations or switches likely to be related to factors that are prognostic for the outcome?	NA
		If N/PN, answer questions relating to baseline confounding (1.4 to 1.6)	
		If Y/PY, answer questions relating to both baseline and time-varying confounding (1.7 and 1.8)	
		1.4. Did the authors use an appropriate analysis method that controlled for all the important confounding domains?	Y
		1.5. If Y/PY to 1.4: Were confounding domains that were controlled for measured validly and reliably by the variables available in this study?	Y
		1.6. Did the authors control for any post-intervention variables that could have been affected by the intervention?	Y
		1.7. Did the authors use an appropriate analysis method that controlled for all the important confounding domains and for time-varying confounding?	Y
		1.8. If Y/PY to 1.7: Were confounding domains that were controlled for measured validly and reliably by the variables available in this study?	Y
		Risk of bias judgement	Moderate
	Bias in selection of participants into the study	2.1. Was selection of participants into the study (or into the analysis) based on participant characteristics observed after the start of intervention?	N
		2.2. If Y/PY to 2.1: Were the post-intervention variables that influenced selection likely to be associated with intervention?	NA
		2.3. If Y/PY to 2.2: Were the post-intervention variables that influenced selection likely to be influenced by the outcome or a cause of the outcome?	NA
		2.4. Do start of follow-up and start of intervention coincide for most participants?	Y
		2.5. If Y/PY to 2.2 and 2.3, or N/PN to 2.4: Were adjustment techniques used that are likely to correct for the presence of selection biases?	NA
		Risk of bias judgement	Low
	Bias in classification of interventions	3.1. Were intervention groups clearly defined?	Y
		3.2. Was the information used to define intervention groups recorded at the start of the intervention?	Y
		3.3. Could classification of intervention status have been affected by knowledge of the outcome or risk of the outcome?	PN
		Risk of bias judgement	Low
	Bias due to deviations from intended interventions	4.1. Were there deviations from the intended intervention beyond what would be expected in usual practice?	N
		4.2. If Y/PY to 4.1: Were these deviations from intended intervention unbalanced between groups and likely to have affected the outcome?	NA
		If your aim for this study is to assess the effect of starting and adhering to intervention, answer questions 4.3 to 4.6	
		4.3. Were important co-interventions balanced across intervention groups?	NA

		4.4. Was the intervention implemented successfully for most participants?	NA
		4.5. Did study participants adhere to the assigned intervention regimen?	NA
		4.6. If N/PN to 4.3, 4.4 or 4.5: Was an appropriate analysis used to estimate the effect of starting and adhering to the intervention?	NA
		Risk of bias judgement	Low
	Bias due to missing data	5.1. Were outcome data available for all, or nearly all, participants?	PN
		5.2. Were participants excluded due to missing data on intervention status?	PY
		5.3. Were participants excluded due to missing data on other variables needed for the analysis?	PN
		5.4. If PN/N to 5.1, or Y/PY to 5.2 or 5.3: Are the proportion of participants and reasons for missing data similar across interventions?	NI
		5.5. If PN/N to 5.1, or Y/PY to 5.2 or 5.3: Is there evidence that results were robust to the presence of missing data?	NI
		Risk of bias judgement	Serious
	Bias in measurement of outcomes	6.1. Could the outcome measure have been influenced by knowledge of the intervention received?	PN
		6.2. Were outcome assessors aware of the intervention received by study participants?	Y
		6.3. Were the methods of outcome assessment comparable across intervention groups?	Y
		6.4. Were any systematic errors in measurement of the outcome related to intervention received?	PN
		Risk of bias judgement	Moderate
	Bias in selection of the reported result	Is the reported effect estimate likely to be selected, on the basis of the results, from...	
		7.1. ... multiple outcome measurements within the outcome domain?	PN
		7.2. ... multiple analyses of the intervention-outcome relationship?	PN
		7.3. ... different subgroups?	PN
		Risk of bias judgement	Low
	Overall bias	Risk of bias judgement	Moderate
Saariaho, Saariaho, Mattila, Joukamaa and Karukivi [129]	Bias due to confounding	1.1 Is there potential for confounding of the effect of intervention in this study?	Y
		If N/PN to 1.1: the study can be considered to be at low risk of bias due to confounding and no further signalling questions need be considered	
		If Y/PY to 1.1: determine whether there is a need to assess time-varying confounding:	
		1.2 Was the analysis based on splitting participants' follow up time according to intervention received?	N
		If N/PN, answer questions relating to baseline confounding (1.4 to 1.6)	
		If Y/PY, go to question 1.3.	
		1.3 Were intervention discontinuations or switches likely to be related to factors that are prognostic for the outcome?	NA
		If N/PN, answer questions relating to baseline confounding (1.4 to 1.6)	
		If Y/PY, answer questions relating to both baseline and time-varying confounding (1.7 and 1.8)	

	1.4. Did the authors use an appropriate analysis method that controlled for all the important confounding domains?	PY
	1.5. If Y/PY to 1.4: Were confounding domains that were controlled for measured validly and reliably by the variables available in this study?	PY
	1.6. Did the authors control for any post-intervention variables that could have been affected by the intervention?	N
	1.7. Did the authors use an appropriate analysis method that controlled for all the important confounding domains and for time-varying confounding?	Y
	1.8. If Y/PY to 1.7: Were confounding domains that were controlled for measured validly and reliably by the variables available in this study?	PY
	Risk of bias judgement	Low
Bias in selection of participants into the study	2.1. Was selection of participants into the study (or into the analysis) based on participant characteristics observed after the start of intervention?	N
	2.2. If Y/PY to 2.1: Were the post-intervention variables that influenced selection likely to be associated with intervention?	NA
	2.3. If Y/PY to 2.2: Were the post-intervention variables that influenced selection likely to be influenced by the outcome or a cause of the outcome?	NA
	2.4. Do start of follow-up and start of intervention coincide for most participants?	Y
	2.5. If Y/PY to 2.2 and 2.3, or N/PN to 2.4: Were adjustment techniques used that are likely to correct for the presence of selection biases?	NA
	Risk of bias judgement	Low
Bias in classification of interventions	3.1. Were intervention groups clearly defined?	PN
	3.2. Was the information used to define intervention groups recorded at the start of the intervention?	PN
	3.3. Could classification of intervention status have been affected by knowledge of the outcome or risk of the outcome?	PN
	Risk of bias judgement	Critical
Bias due to deviations from intended interventions	4.1. Were there deviations from the intended intervention beyond what would be expected in usual practice?	N
	4.2. If Y/PY to 4.1: Were these deviations from intended intervention unbalanced between groups and likely to have affected the outcome?	NA
	If your aim for this study is to assess the effect of starting and adhering to intervention, answer questions 4.3 to 4.6	
	4.3. Were important co-interventions balanced across intervention groups?	NA
	4.4. Was the intervention implemented successfully for most participants?	NA
	4.5. Did study participants adhere to the assigned intervention regimen?	NA
	4.6. If N/PN to 4.3, 4.4 or 4.5: Was an appropriate analysis used to estimate the effect of starting and adhering to the intervention?	NA
	Risk of bias judgement	Low
Bias due to missing data	5.1. Were outcome data available for all, or nearly all, participants?	N
	5.2. Were participants excluded due to missing data on intervention status?	Y

		5.3. Were participants excluded due to missing data on other variables needed for the analysis?	N
		5.4. If PN/N to 5.1, or Y/PY to 5.2 or 5.3: Are the proportion of participants and reasons for missing data similar across interventions?	PY
		5.5. If PN/N to 5.1, or Y/PY to 5.2 or 5.3: Is there evidence that results were robust to the presence of missing data?	Y
		Risk of bias judgement	Moderate
	Bias in measurement of outcomes	6.1. Could the outcome measure have been influenced by knowledge of the intervention received?	PN
		6.2. Were outcome assessors aware of the intervention received by study participants?	Y
		6.3. Were the methods of outcome assessment comparable across intervention groups?	Y
		6.4. Were any systematic errors in measurement of the outcome related to intervention received?	PN
		Risk of bias judgement	Moderate
	Bias in selection of the reported result	Is the reported effect estimate likely to be selected, on the basis of the results, from...	
		7.1. ... multiple outcome measurements within the outcome domain?	PN
		7.2. ... multiple analyses of the intervention-outcome relationship?	PN
		7.3. ... different subgroups?	PY
		Risk of bias judgement	Moderate
	Overall bias	Risk of bias judgement	Moderate
Saariaho, Saariaho, Mattila, Ohtonen, Joukamaa and Karukivi [130]	Bias due to confounding	1.1 Is there potential for confounding of the effect of intervention in this study?	Y
		If N/PN to 1.1: the study can be considered to be at low risk of bias due to confounding and no further signalling questions need be considered	
		If Y/PY to 1.1: determine whether there is a need to assess time-varying confounding:	
		1.2 Was the analysis based on splitting participants' follow up time according to intervention received?	N
		If N/PN, answer questions relating to baseline confounding (1.4 to 1.6)	
		If Y/PY, go to question 1.3.	
		1.3 Were intervention discontinuations or switches likely to be related to factors that are prognostic for the outcome?	NA
		If N/PN, answer questions relating to baseline confounding (1.4 to 1.6)	
		If Y/PY, answer questions relating to both baseline and time-varying confounding (1.7 and 1.8)	
		1.4. Did the authors use an appropriate analysis method that controlled for all the important confounding domains?	PY
		1.5. If Y/PY to 1.4: Were confounding domains that were controlled for measured validly and reliably by the variables available in this study?	PY
		1.6. Did the authors control for any post-intervention variables that could have been affected by the intervention?	N
		1.7. Did the authors use an appropriate analysis method that controlled for all the important confounding domains and for time-varying confounding?	Y
		1.8. If Y/PY to 1.7: Were confounding domains that were controlled for measured validly and reliably by the variables available in this study?	PY

	Risk of bias judgement	Low
Bias in selection of participants into the study	2.1. Was selection of participants into the study (or into the analysis) based on participant characteristics observed after the start of intervention?	N
	2.2. If Y/PY to 2.1: Were the post-intervention variables that influenced selection likely to be associated with intervention?	NA
	2.3. If Y/PY to 2.2: Were the post-intervention variables that influenced selection likely to be influenced by the outcome or a cause of the outcome?	NA
	2.4. Do start of follow-up and start of intervention coincide for most participants?	PY
	2.5. If Y/PY to 2.2 and 2.3, or N/PN to 2.4: Were adjustment techniques used that are likely to correct for the presence of selection biases?	NA
	Risk of bias judgement	Low
Bias in classification of interventions	3.1. Were intervention groups clearly defined?	PN
	3.2. Was the information used to define intervention groups recorded at the start of the intervention?	PN
	3.3. Could classification of intervention status have been affected by knowledge of the outcome or risk of the outcome?	PN
	Risk of bias judgement	Critical
Bias due to deviations from intended interventions	4.1. Were there deviations from the intended intervention beyond what would be expected in usual practice?	PN
	4.2. If Y/PY to 4.1: Were these deviations from intended intervention unbalanced between groups and likely to have affected the outcome?	NA
	If your aim for this study is to assess the effect of starting and adhering to intervention, answer questions 4.3 to 4.6	
	4.3. Were important co-interventions balanced across intervention groups?	NA
	4.4. Was the intervention implemented successfully for most participants?	NA
	4.5. Did study participants adhere to the assigned intervention regimen?	NA
	4.6. If N/PN to 4.3, 4.4 or 4.5: Was an appropriate analysis used to estimate the effect of starting and adhering to the intervention?	NA
	Risk of bias judgement	Low
Bias due to missing data	5.1. Were outcome data available for all, or nearly all, participants?	N
	5.2. Were participants excluded due to missing data on intervention status?	Y
	5.3. Were participants excluded due to missing data on other variables needed for the analysis?	N
	5.4. If PN/N to 5.1, or Y/PY to 5.2 or 5.3: Are the proportion of participants and reasons for missing data similar across interventions?	PY
	5.5. If PN/N to 5.1, or Y/PY to 5.2 or 5.3: Is there evidence that results were robust to the presence of missing data?	Y
	Risk of bias judgement	Moderate
Bias in measurement of outcomes	6.1. Could the outcome measure have been influenced by knowledge of the intervention received?	PY
	6.2. Were outcome assessors aware of the intervention received by study participants?	Y
	6.3. Were the methods of outcome assessment comparable across intervention groups?	Y

		6.4. Were any systematic errors in measurement of the outcome related to intervention received?	N
		Risk of bias judgement	Moderate
	Bias in selection of the reported result	Is the reported effect estimate likely to be selected, on the basis of the results, from...	
		7.1. ... multiple outcome measurements within the outcome domain?	PN
		7.2. ... multiple analyses of the intervention-outcome relationship?	PN
		7.3. ... different subgroups?	PY
		Risk of bias judgement	Moderate
	Overall bias	Risk of bias judgement	Moderate
Porcelli, Bagby, Taylor, De Carne, Leandro and Todarello [131]	Bias due to confounding	1.1 Is there potential for confounding of the effect of intervention in this study?	Y
		If N/PN to 1.1: the study can be considered to be at low risk of bias due to confounding and no further signalling questions need be considered	
		If Y/PY to 1.1: determine whether there is a need to assess time-varying confounding:	
		1.2 Was the analysis based on splitting participants' follow up time according to intervention received?	N
		If N/PN, answer questions relating to baseline confounding (1.4 to 1.6)	
		If Y/PY, go to question 1.3.	
		1.3 Were intervention discontinuations or switches likely to be related to factors that are prognostic for the outcome?	NA
		If N/PN, answer questions relating to baseline confounding (1.4 to 1.6)	
		If Y/PY, answer questions relating to both baseline and time-varying confounding (1.7 and 1.8)	
		1.4. Did the authors use an appropriate analysis method that controlled for all the important confounding domains?	Y
		1.5. If Y/PY to 1.4: Were confounding domains that were controlled for measured validly and reliably by the variables available in this study?	PY
		1.6. Did the authors control for any post-intervention variables that could have been affected by the intervention?	N
		1.7. Did the authors use an appropriate analysis method that controlled for all the important confounding domains and for time-varying confounding?	Y
		1.8. If Y/PY to 1.7: Were confounding domains that were controlled for measured validly and reliably by the variables available in this study?	PY
		Risk of bias judgement	Moderate
	Bias in selection of participants into the study	2.1. Was selection of participants into the study (or into the analysis) based on participant characteristics observed after the start o intervention?	N
		2.2. If Y/PY to 2.1: Were the post-intervention variables that influenced selection likely to be associated with intervention?	NA
		2.3. If Y/PY to 2.2: Were the post-intervention variables that influenced selection likely to be influenced by the outcome or a cause of the outcome?	NA
		2.4. Do start of follow-up and start of intervention coincide for most participants?	Y

	2.5. If Y/PY to 2.2 and 2.3, or N/PN to 2.4: Were adjustment techniques used that are likely to correct for the presence of selection biases?	NA
	Risk of bias judgement	Low
Bias in classification of interventions	3.1. Were intervention groups clearly defined?	Y
	3.2. Was the information used to define intervention groups recorded at the start of the intervention?	Y
	3.3. Could classification of intervention status have been affected by knowledge of the outcome or risk of the outcome?	PN
	Risk of bias judgement	Low
Bias due to deviations from intended interventions	4.1. Were there deviations from the intended intervention beyond what would be expected in usual practice?	PN
	4.2. If Y/PY to 4.1: Were these deviations from intended intervention unbalanced between groups and likely to have affected the outcome?	NA
	If your aim for this study is to assess the effect of starting and adhering to intervention, answer questions 4.3 to 4.6	
	4.3. Were important co-interventions balanced across intervention groups?	NA
	4.4. Was the intervention implemented successfully for most participants?	NA
	4.5. Did study participants adhere to the assigned intervention regimen?	NA
	4.6. If N/PN to 4.3, 4.4 or 4.5: Was an appropriate analysis used to estimate the effect of starting and adhering to the intervention?	NA
	Risk of bias judgement	Low
Bias due to missing data	5.1. Were outcome data available for all, or nearly all, participants?	PN
	5.2. Were participants excluded due to missing data on intervention status?	PY
	5.3. Were participants excluded due to missing data on other variables needed for the analysis?	PN
	5.4. If PN/N to 5.1, or Y/PY to 5.2 or 5.3: Are the proportion of participants and reasons for missing data similar across interventions?	PY
	5.5. If PN/N to 5.1, or Y/PY to 5.2 or 5.3: Is there evidence that results were robust to the presence of missing data?	NI
	Risk of bias judgement	Moderate
Bias in measurement of outcomes	6.1. Could the outcome measure have been influenced by knowledge of the intervention received?	PN
	6.2. Were outcome assessors aware of the intervention received by study participants?	Y
	6.3. Were the methods of outcome assessment comparable across intervention groups?	Y
	6.4. Were any systematic errors in measurement of the outcome related to intervention received?	PN
	Risk of bias judgement	Moderate
Bias in selection of the reported result	Is the reported effect estimate likely to be selected, on the basis of the results, from...	
	7.1. ... multiple outcome measurements within the outcome domain?	PN
	7.2. ... multiple analyses of the intervention-outcome relationship?	PN
	7.3. ... different subgroups?	Y

	Risk of bias judgement	Moderate
Overall bias	Risk of bias judgement	Moderate

Y: yes; PY: probably yes; PN: probably no; N: no; NI: no information; NA: no answer.
